# Association between systemic inflammation and long-term mortality in emergency department patients with severe hypertension

**DOI:** 10.1016/j.clinsp.2026.101186

**Published:** 2026-10-03

**Authors:** Byungwoo Yoo, Byung Sik Kim, Hyun-Jin Kim, Jeong-Hun Shin

**Affiliations:** a Hanyang University College of Medicine, Seoul, Republic of Korea; b Division of Cardiology, Department of Internal Medicine, Hanyang University College of Medicine, Hanyang University Guri Hospital, Guri, Republic of Korea

**Keywords:** Inflammation, Hypertension, Severe hypertension, Emergency department, Mortality

## Abstract

**Background:**

The prognostic role of systemic inflammatory indicators in patients with severe hypertension remains unclear. This study evaluated the associations between several inflammatory markers, including Neutrophil-to-Lymphocyte Ratio (NLR), Lymphocyte-to-Monocyte Ratio (LMR), Platelet-to-Lymphocyte Ratio (PLR), Systemic Immune-Inflammation Index (SII), C-Reactive Protein (CRP) level, and long-term mortality.

**Methods:**

This retrospective cohort included 5,320 adult patients presenting to the emergency department with severe hypertension (systolic blood pressure ≥ 180 mmHg or diastolic blood pressure ≥ 110 mmHg) between 2016 and 2019. All patients underwent complete blood counts and CRP testing. Patients were categorized into tertiles for each inflammatory marker.

**Results:**

Higher tertiles of NLR, PLR, SII, and CRP were associated with increased 3-year all-cause mortality, while higher LMR was associated with lower mortality. Adjusted hazard ratios (95% Confidence Intervals) comparing the highest to lowest tertiles were: NLR 1.61 (1.35–1.92), LMR 0.71 (0.60–0.85), PLR 1.22 (1.03–1.44), SII 1.57 (1.33–1.86), and CRP 2.21 (1.84–2.65). Dose–response analyses revealed linear associations between NLR and SII, an inverse J-shaped pattern for LMR, a J-shaped curve for PLR, and a nonlinear positive association for CRP. CRP showed the highest discriminatory performance among the evaluated markers.

**Conclusion:**

Systemic inflammatory indicators were associated with long-term mortality in patients with severe hypertension. These findings suggest that inflammatory markers may provide prognostic information in this population, although their role in risk stratification and follow-up planning requires further evaluation.

## Highlights

• Inflammatory markers were associated with 3-year mortality in severe hypertension.
• NLR and SII showed linear associations; LMR, PLR, and CRP were nonlinear.
• CRP showed the highest AUC, but overall discrimination was modest.

## Introduction

Severe hypertension, an acute and severe manifestation of hypertension, is characterized by a sudden and marked elevation in Blood Pressure (BP) and occurs in approximately 1%–2% of individuals with hypertension.^[^1, 2, 3^]^ Despite advances in antihypertensive therapies over recent decades, severe hypertension continues to pose significant clinical challenges.^[^^1^^,^^4^^]^ The prognosis is particularly poor when hypertension-mediated organ damage (HMOD) occurs because it is strongly associated with increased cardiovascular morbidity and mortality.^[^5, 6, 7^]^ However, data on the prognostic factors of severe hypertension remain limited, highlighting the need for better risk stratification and the prediction of long-term outcomes.

Systemic inflammation is a key contributor to the development and progression of hypertension and is associated with HMOD.^[^^8^^]^ In severe hypertension, inflammatory activation has been reported to be accentuated and to correlate with the presence of acute HMOD.^[^^9^^]^ Inflammatory indices derived from routine blood cell counts, such as the Neutrophil-to-Lymphocyte Ratio (NLR), Lymphocyte-to-Monocyte Ratio (LMR), Platelet-to-Lymphocyte Ratio (PLR), and Systemic Immune-Inflammation Index (SII), have been increasingly studied as simple, cost-effective, and accessible markers.^[^^10^^]^ Evidence is accumulating regarding the association between these systemic inflammatory markers and various clinical conditions, including hypertension,^[^^11^^]^ cardiovascular disease,^[^^10^^]^ arrhythmia,^[^^12^^]^ cancer,^[^^13^^]^ and premature death.^[^^14^^]^ Although these inflammatory markers have been studied in population-based and cardiovascular cohorts, their associations with long-term outcomes in patients with severe hypertension remain unclear. To address these gaps, we evaluated the association of systemic inflammatory indicators, including NLR, LMR, PLR, SII, and C-Reactive Protein (CRP) level, with long-term all-cause mortality and compared their predictive performance in patients with severe hypertension.

## Methods

### Study participants

This retrospective cohort study was conducted at a single regional emergency medical center affiliated with a university hospital in Korea. From 172,105 Emergency Department (ED) visits between January 1, 2016, and December 31, 2019, we identified 10,083 individuals with Systolic BP (SBP) ≥ 180 mmHg or Diastolic BP (DBP) ≥ 110 mmHg. Patients were excluded if they were younger than 18-years, presented with an acute traumatic injury, or visited solely for medical certification. Only the data from the first visit were included for individuals who visited the ED multiple times during the study period. After applying these criteria, 6,467 patients with severe hypertension were identified. Of these, 5,320 patients with available laboratory data necessary for calculating systemic inflammatory indices were included in the final analysis (Fig. 1).

**Fig. 1:**
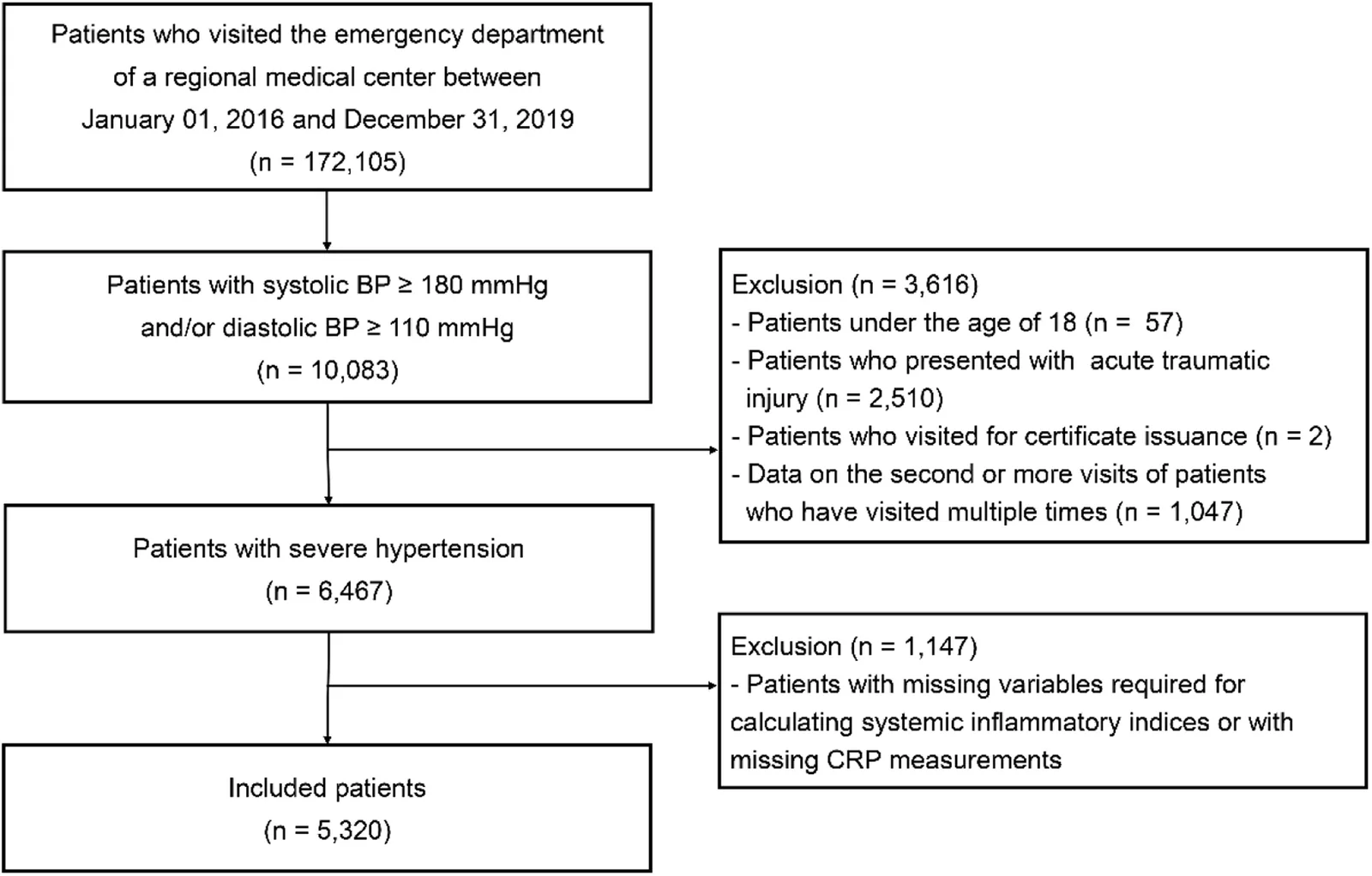
Flowchart of the study population. BP, Blood Pressure.

### Data collection and definitions

Eligible patients were initially identified from the National Emergency Department Information System (NEDIS), a mandatory nationwide emergency care database in Korea that records information on all patients visiting emergency medical institutions, including initial vital signs and outcomes of the index ED visit. Additional clinical information, including laboratory data, diagnostic test findings, comorbidities, and acute HMOD status, was extracted from the hospital electronic medical records. Data abstraction was performed by trained research staff using predefined variable definitions and a standardized data abstraction form based on the protocol used in previous studies from this cohort.^[^^4^^,^^6^^,^^15^^]^ Several meetings were held between the research staff and the principal investigator to review variable definitions and resolve ambiguous or discrepant cases. Chart abstractors were not involved in the statistical analyses, and inflammatory marker tertiles were generated after completion of data extraction. Mortality status and date of death were obtained from the National Health Insurance Service database and were not determined by chart reviewers.

The collected information included demographic characteristics, initial BP, laboratory data, and cardiovascular comorbidities, such as hypertension, diabetes mellitus, dyslipidemia, Chronic Kidney Disease (CKD), end-stage renal disease, ischemic or hemorrhagic stroke, heart failure, and coronary artery disease. Initial BP was defined as the first BP value recorded in NEDIS at ED presentation. BP was measured over the brachial artery using an automated BP machine (Spot Vital Signs LXi; Welch Allyn, Skaneateles Falls, NY, USA) as part of routine ED care. Laboratory data included hemoglobin levels, estimated Glomerular Filtration Rate (eGFR), troponin-I levels, and complete blood cell counts, including neutrophil, lymphocyte, monocyte, and platelet counts. Diagnostic tests such as urinalysis, chest radiography, and Electrocardiography (ECG) were also reviewed. Cardiomegaly was defined as a cardiothoracic ratio greater than 0.5 on chest radiography. Left Ventricular Hypertrophy (LVH) was defined based on ECG criteria using either the Sokolow-Lyon criterion (sum of the S wave in lead V1 and the R wave in V5 or V6 ≥3.5 mV) or the Cornell voltage criterion (sum of the R wave in aVL and the S or QS wave in V3 exceeding 2.8 mV in men or 2.0 mV in women).

Patients with severe hypertension were categorized as hypertensive emergencies in the presence of acute HMOD, and as hypertensive urgencies otherwise.^[^^16^^,^^17^^]^ Acute HMOD was defined as the presence of acute heart failure, ischemic stroke, hypertensive encephalopathy, intracerebral hemorrhage, hypertensive retinopathy, acute coronary syndrome, acute kidney injury, or aortic dissection. Concomitant infection at the index ED visit was identified based on the discharge diagnosis documented in the ED medical record. The outcomes recorded during the index visit and follow-up included hospital admission, discharge, ED revisits, and all-cause mortality. Data on mortality and the date of death were obtained from the National Health Insurance Service of South Korea.

### Systemic inflammatory indicators

Peripheral blood samples were obtained during the index ED visit as part of routine clinical evaluation. For inflammatory markers, we used the first available complete blood count and CRP measurement obtained during the index ED visit. These blood samples were generally obtained during the initial ED evaluation after the first BP measurement and before disposition decisions. When multiple laboratory measurements were available during the index ED visit, the earliest values were used. Neutrophil, lymphocyte, monocyte, and platelet counts were measured using an automated hematology analyzer (DxH 800; Beckman Coulter, CA, USA). Based on these values, systemic inflammatory indicators were calculated as follows: NLR, neutrophils/lymphocytes; LMR, lymphocytes/monocytes; PLR, platelets/lymphocytes; and SII as (neutrophils × platelets)/lymphocytes. Serum CRP levels were measured via an immunoturbidimetric assay (AU5800; Beckman Coulter, Brea, CA, USA). These indices have been widely investigated in previous studies as accessible and reliable markers of systemic inflammation in diverse clinical settings.^[^^10^^,^^12^^]^

### Ethical consideration

The study protocol adhered to the ethical principles outlined in the Declaration of Helsinki and was approved by the Institutional Review Board of the Hanyang University Guri Hospital (IRB. 2026-05-004). The Institutional Review Board waived the requirement for written informed consent because of the retrospective nature of the study.

### Statistical analysis

Continuous variables were presented as means with standard deviations, and categorical variables were expressed as numbers with percentages. For comparisons among the three groups, the distribution of each continuous variable was evaluated using the Kolmogorov-Smirnov test, along with assessments of skewness and kurtosis. Variables with a normal distribution were analyzed using one-way analysis of variance, followed by Tukey’s post-hoc test. For non-normally distributed variables, the Kruskal-Wallis test was used, followed by Dunn’s multiple comparison test. Categorical variables were compared using the Chi-Square test or Fisher’s exact test, as appropriate.

Survival probabilities according to the levels of systemic inflammatory indicators were estimated using Kaplan-Meier curves, and differences between groups were assessed using the log-rank test. The association between each systemic inflammatory indicator and the 3-year all-cause mortality was examined using Cox proportional hazards regression models. In addition to univariate analyses, three multivariate models were constructed to account for potential confounding factors. Model 1 included age, sex, and BP. Model 2 was further adjusted for comorbidities including hypertension, diabetes mellitus, dyslipidemia, ischemic stroke, hemorrhagic stroke, coronary artery disease, heart failure, and CKD. Model 3 additionally incorporated markers of subclinical HMOD, including eGFR, hemoglobin level, cardiomegaly on chest radiography, and electrocardiographic findings such as LVH, myocardial ischemia, and atrial fibrillation. HRs and 95% CIs were calculated for all models. Missing data were assessed for covariates included in the multivariable Cox regression models. The primary Cox regression analyses were performed using complete-case analysis, and the fully adjusted model included 4,402 patients with complete covariate data. The frequency of missing data for model covariates is summarized in Supplemental Table S1. As a sensitivity analysis to assess the robustness of the primary complete-case analyses, multiple imputation by chained equations was performed for missing covariate data among the 5,320 patients included in the final study cohort. The distributions of covariates before and after multiple imputation are summarized in Supplemental Table S2. Cox proportional hazards regression analyses were repeated across the imputed datasets, and estimates were pooled using Rubin’s rules.^[^^18^^]^ Additional sensitivity analyses further adjusted Model 3 for smoking status, Body Mass Index (BMI), or both. These variables were not included in the primary models because of substantial missing data. The primary analysis was also repeated after excluding patients with an infection diagnosis at the index ED visit. Restricted cubic spline analyses were performed to explore potential nonlinear associations between continuous inflammatory indicators and mortality risk. Finally, the discriminatory performance of each inflammatory marker in predicting the 3-year all-cause mortality was compared using the area under the curve derived from time-dependent receiver operating characteristic analyses. All statistical analyses were conducted using the open-source statistical software R (version 4.4.0, www.R-project.org) and RStudio (version 2023.12.1, www.rstudio.com), along with statistical packages including ggplot2, timeROC, rms, survminer, survival, and mice. All tests were two-sided, and a p-value < 0.05 was considered significant.

## Results

### Baseline characteristics

A total of 5,320 patients were included, with a median follow-up of 2.86-years (interquartile range, 1.77–3.93). The baseline characteristics according to NLR tertiles are presented in Table 1. The cohort had a mean age of 62.5±16.6 years, with 49.0% women. Patients in the highest NLR tertile (NLR > 4.39) were older (65.6±17.1 years vs. 61.9±16.8 vs. 60.2±15.4, p < 0.001), but the proportion of women was similar across tertiles (47.9% vs. 48.7% vs. 50.3%, p = 0.347). A higher NLR was associated with an increased prevalence of cardiovascular risk factors (hypertension and diabetes mellitus) and comorbidities (ischemic stroke, heart failure, CKD, end-stage renal disease; all p < 0.001). SBP and DBP were lower in the highest NLR tertile (SBP: 189.3±22.2 mmHg vs. 191.6±22.0 vs. 191.5±22.2, p = 0.002; DBP: 106.4±18.3 mmHg vs. 107.5±17.4 vs. 109.0±16.7, p < 0.001). Laboratory findings showed lower eGFR, lower hemoglobin level, and higher BNP and troponin-I levels in the highest NLR tertile. Inflammatory markers, including neutrophil counts, PLR, SII, and CRP levels were elevated, whereas lymphocyte counts and LMR were lower in the highest tertile (all p < 0.001). Proteinuria, cardiomegaly, and electrocardiographic abnormalities (left ventricular hypertrophy and atrial fibrillation) were more prevalent with an increasing NLR.

**Table 1:** Baseline characteristics according to tertiles of neutrophil-to-lymphocyte ratio.

	**All patients (n = 5,320)**	**Neutrophil-to-lymphocyte ratio**			**p-value**
		Tertile 1*(n = 1,774)	**Tertile 2**†**(n = 1,773)**	**Tertile 3**‡**(n = 1,773)**	
**Age, mean**	62.54 ± 16.60	60.16 ± 15.43	61.92 ± 16.78^a^	65.55 ± 17.10^a^^,^^b^	<0.001
**Women, n (%)**	2607 (49.0)	893 (50.3)	864 (48.7)	850 (47.9)	0.347
**Medical history, n (%)**					
Hypertension	2859 (55.1)	874 (50.4)	955 (55.4)	1030 (59.4)	<0.001
Diabetes mellitus	1429 (27.8)	352 (20.5)	502 (29.4)	575 (33.5)	<0.001
Dyslipidemia	519 (10.2)	182 (10.6)	191 (11.3)	146 (8.6)	0.026
Ischemic stroke	430 (8.4)	97 (5.7)	136 (8.1)	197 (11.5)	<0.001
Hemorrhagic stroke	150 (2.9)	50 (2.9)	41 (2.4)	59 (3.5)	0.199
Coronary artery disease	496 (9.7)	161 (9.4)	185 (10.9)	150 (8.8)	0.091
Heart failure	227 (4.5)	40 (2.3)	77 (4.6)	110 (6.5)	<0.001
Chronic kidney disease	446 (8.7)	67 (3.9)	139 (8.2)	240 (14.1)	<0.001
End-stage renal disease	216 (4.2)	35 (2.0)	64 (3.8)	117 (6.9)	<0.001
**Social history, n (%)**					
Cigarette smoking	922 (25.7)	313 (27.7)	323 (27.1)	286 (22.6)	0.022
Alcohol consumption	1,274 (35.0)	481 (41.5)	422 (35.2)	371 (28.8)	<0.001
**Triage vitals, mean**					
SBP, mmHg	190.79 ± 22.14	191.46 ± 22.16	191.64 ± 21.99	189.25 ± 22.20^a^^,^^b^	0.002
DBP, mmHg	107.64 ± 17.48	109.02 ± 16.70	107.54 ± 17.36^a^	106.36 ± 18.26^a^	<0.001
**Laboratory tests**					
eGFR, mL/min/1.73 m^2^	79.44 ± 30.35	86.22 ± 24.73	81.22 ± 29.95^a^	70.86 ± 33.67^a^^,^^b^	<0.001
Hb, g/dL	13.53 ± 2.17	13.91 ± 1.87	13.59 ± 2.09^a^	13.09 ± 2.43^a^^,^^b^	<0.001
BNP, pg/mL	378.6 ± 783.6	242.9 ± 609.6	318.8 ± 661.8^a^	558.0 ± 973.9^a^^,^^b^	<0.001^d^
Troponin-I, ng/mL	0.18 ± 2.41	0.04 ± 0.22	0.14 ± 1.36^a^	0.36 ± 3.93^a^^,^^b^	<0.001^d^
Neutrophil count, 10^^9^ cells/L	6.41 ± 3.93	3.85 ± 1.51	5.69 ± 2.16^a^	9.69 ± 4.64^a^^,^^b^	<0.001^d^
Lymphocyte count, 10^^9^ cells/L	2.03 ± 2.05	3.08 ± 3.13	1.91 ± 0.71^a^	1.10 ± 0.52^a^^,^^b^	<0.001^d^
Monocyte count, 10^^9^ cells/L	0.59 ± 0.89	0.58 ± 0.25	0.62 ± 1.48	0.58 ± 0.32	0.125^d^
Platelet count, 10^^9^ cells/L	224.07 ± 76.04	224.21 ± 67.72	223.25 ± 72.51	224.76 ± 86.65	0.218^d^
**Urinary analysis**					
Proteinuria^c^, n (%)	1155 (33.0)	241 (21.7)	342 (29.9)	572 (45.8)	<0.001
Chest radiography					
Cardiomegaly, n (%)	711 (14.1)	203 (12.1)	237 (14.2)	271 (16.0)	0.006
**Electrocardiography**					
LVH, n (%)	576 (12.3)	161 (10.3)	192 (12.3)	223 (14.2)	0.004
Myocardial ischemia, n (%)	318 (6.8)	97 (6.2)	100 (6.4)	121 (7.7)	0.196
Atrial fibrillation, n (%)	259 (5.5)	71 (4.5)	85 (5.4)	103 (6.6)	0.047
Acute HMOD, n (%)	1859 (34.9)	636 (35.9)	609 (34.3)	614 (34.6)	0.608
**Systemic inflammation indicators**					
Neutrophil-to-lymphocyte ratio	5.40 ± 8.85	1.36 ± 0.40	3.04 ± 0.66^a^	11.80 ± 13.09^a^^,^^b^	<0.001^d^
Lymphocyte-to-monocyte ratio	3.99 ± 3.31	5.67 ± 3.89	3.68 ± 2.05^a^	2.63 ± 2.97^a^^,^^b^	<0.001^d^
Platelet-to-lymphocyte ratio	156.10 ± 154.18	83.46 ± 33.16	126.64 ± 50.12^a^	258.23 ± 226.16^a^^,^^b^	<0.001^d^
Systemic immune-inflammation index	1225.97 ± 2515.86	306.40 ± 132.07	677.54 ± 267.86^a^	2694.48 ± 3950.30^a^^,^^b^	<0.001^d^
C-reactive protein, mg/dL	1.67 ± 4.64	0.40 ± 1.35	1.00 ± 2.68^a^	3.62 ± 7.06^a^^,^^b^	<0.001^d^

Data are presented as n (%) or mean ± SD, as appropriate. BNP, B-type Natriuretic Peptide; DBP, Diastolic Blood Pressure; eGFR, Estimated Glomerular Filtration Rate; Hb, Hemoglobin; HMOD, Hypertension-Mediated Organ Damage; LVH, Left Ventricular Hypertrophy; NLR, Neutrophil-to-Lymphocyte Ratio; SD, Standard Deviation; SBP, Systolic Blood Pressure. Percentages were calculated using the number of patients with available data for each variable as the denominator.

⁎ The range of NLR in tertile 1 is < 2.04.

† The range of NLR in tertile 2 is 2.04–4.39.

‡ The range of NLR in tertile 3 is > 4.39.

a Post hoc p: Significant difference, p < 0.05, compared with the first tertile.

b Post hoc p: Significant difference, p < 0.05, compared with the second tertile.

c Proteinuria was defined as dipstick urinalysis result ≥ 1+.

d Assessed by nonparametric test.

Baseline characteristics stratified by PLR, SII, and CRP tertiles generally showed trends similar to those of NLR, with higher tertiles associated with older age, greater comorbidity burden, worse renal function, and elevated cardiac and inflammatory marker levels. In contrast, LMR showed the opposite pattern, with higher LMR tertiles reflecting more favorable clinical profiles (Supplemental Tables S3–6).

### Outcomes of the index visit and follow-up

Among the 5,320 patients, 2,818 (53.0%) were admitted, and 6 (0.1%) died in the ED. Patients in the highest NLR tertile were more likely to be admitted than those in the lower tertiles (62.8% vs. 49.8% and 46.3%, respectively; p < 0.001). The rates of ED revisits were generally similar across groups, which was higher in patients in the middle tertile. In contrast, the 1-month, 3-month, 1-year, and 3-year all-cause mortality rates progressively increased with higher NLR (Table 2). Consistent trends were observed for other systemic inflammatory indicators. Higher tertiles of PLR, SII, and CRP were associated with increased risks of admission and all-cause mortality across all time points. In contrast, lower LMR levels were associated with worse outcomes, including higher admission and mortality rates (Supplemental Table S7).

**Table 2:** Outcomes of the index visit to the emergency department and during the follow-up period according to tertiles of neutrophil-to-lymphocyte ratio.

	**All patients** **(n = 5,320)**	**Tertile 1**^a^ **(n = 1,774)**	**Tertile 2**^b^ **(n = 1,773)**	**Tertile 3**^c^ **(n = 1,773)**	**p-value**
**Outcomes of the index visit to the ED, n (%)**					
Admission	2818 (53.0)	821 (46.3)	883 (49.8)	1114 (62.8)	<0.001
Discharge	1971 (37.0)	764 (43.1)	703 (39.7)	504 (28.4)	<0.001
Discharge against medical advice	525 (9.9)	188 (10.6)	186 (10.5)	151 (8.5)	0.065
Death in the emergency department	6 (0.1)	1 (0.1)	1 (0.1)	4 (0.2)	0.223
Revisit to ED, n (%)					
1-month revisit	380 (7.1)	124 (7.0)	139 (7.8)	117 (6.6)	0.341
3-months revisit	695 (13.1)	235 (13.2)	236 (13.3)	224 (12.6)	0.804
1-year revisit	1,210 (22.7)	398 (22.4)	424 (23.9)	388 (21.9)	0.329
Mortality, n (%)					
1-month mortality	216 (4.1)	45 (2.5)	47 (2.7)	124 (7.0)	<0.001
3-months mortality	331 (6.2)	68 (3.8)	79 (4.5)	184 (10.4)	<0.001
1-year mortality	620 (11.7)	126 (7.1)	164 (9.2)	330 (18.6)	<0.001
3-year mortality	965 (18.1)	204 (11.5)	263 (14.8)	498 (28.1)	<0.001

Data are presented as n (%). ED, emergency department; NLR, neutrophil-to-lymphocyte ratio.

a The range of NLR in tertile 1 is < 2.04.

b The range of NLR in tertile 2 is 2.04–4.39.

c The range of NLR in tertile 3 is > 4.39.

The Kaplan-Meier survival curves for all-cause mortality demonstrated a stepwise increase in mortality with higher tertiles of NLR (Fig. 2). This association remained consistent between patients with and without acute HMOD. Similar trends were observed for PLR, SII, and CRP, whereas LMR showed an inverse relationship, with the most favorable survival in the highest tertiles (Supplemental Fig. S1–4). In multivariable Cox regression models (Table 3), higher NLR was associated with an increased risk of all-cause mortality in the overall population, with a fully adjusted Hazard Ratio (HR) of 1.61 (95% Confidence Interval [95% CI: 1.35–1.92]) for the highest tertile compared to the lowest. This association was consistently observed in patients with acute HMOD (HR = 1.96, 95% CI: 1.55–2.48) and without acute HMOD (HR = 1.44, 95% CI: 1.10–1.88). Similarly, the highest tertiles of PLR (HR = 1.22, 95% CI: 1.03–1.44), SII (HR = 1.57, 95% CI: 1.33–1.86), and CRP (HR = 2.21, 95% CI: 1.84–2.65) were significantly associated with increased mortality risk. In contrast, a higher LMR was associated with a reduced risk of mortality (HR = 0.71, 95% CI: 0.60–0.85 for the highest vs. lowest tertile in the overall population), with similar inverse associations observed in both patients with and without acute HMOD. The associations between systemic inflammatory indicators and 3-year all-cause mortality were generally consistent in sensitivity analyses using multiple imputation for missing covariate data in the overall cohort (Supplemental Table S8). The effect estimates were also generally consistent in sensitivity analyses further adjusting for smoking status, BMI, or both variables (Supplemental Table S9). Excluding patients with an infection diagnosis at the index ED visit also did not materially alter the associations (Supplemental Table S10).

**Fig. 2:**
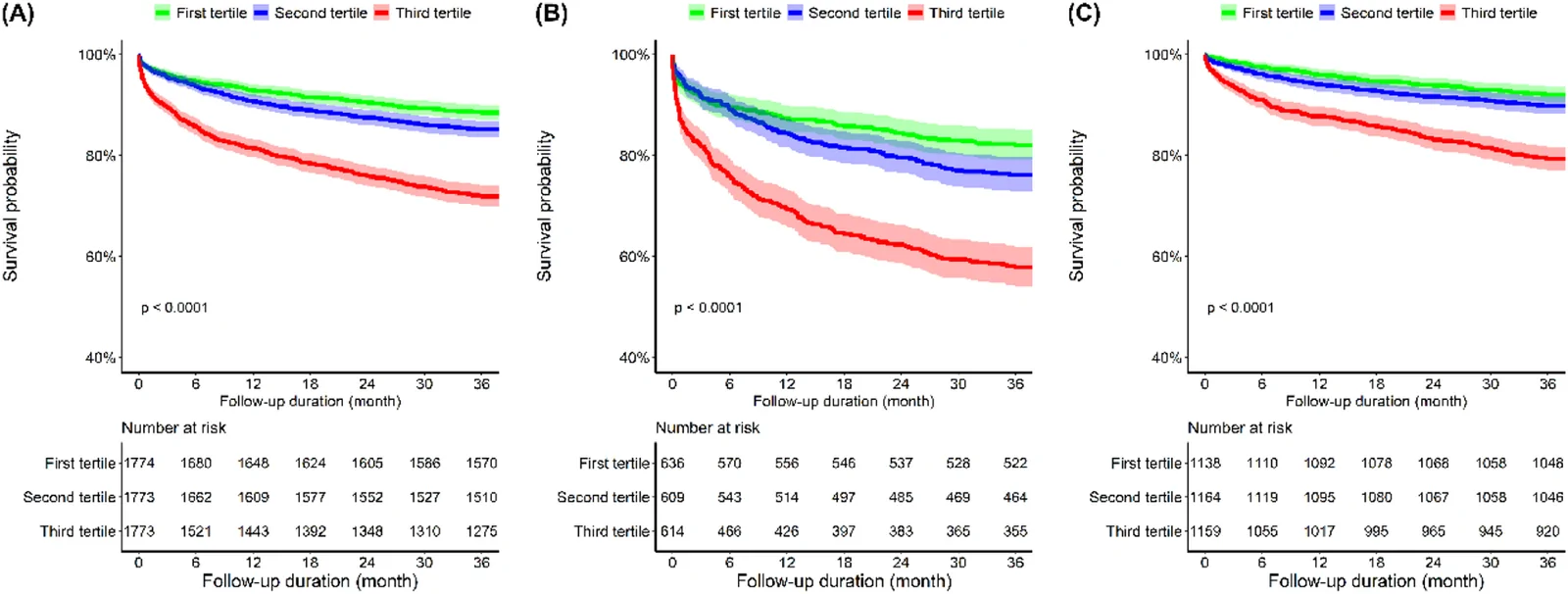
Kaplan-Meier curves for survival probability according to neutrophil-to-lymphocyte ratio tertiles in (A) overall patients, (B) patients with acute HMOD, and (C) patients without acute HMOD. HMOD, Hypertension-Mediated Organ Damage.

**Table 3:** Hazard ratios for 3-year all-cause mortality according to tertiles of NLR, LMR, PLR, SII, and CRP.

**Neutrophil-to-lymphocyte ratio**	**Unadjusted HR (95% CI)**	**Model 1**^a^**(95% CI)**	**Model 2**^b^**(95% CI)**	**Model 3**^c^**(95% CI)**
All patients				
Tertile 1	REF	REF	REF	REF
Tertile 2	1.32 (1.10–1.58)	1.15 (0.96–1.38)	1.08 (0.90–1.31)	1.06 (0.88–1.29)
Tertile 3	2.72 (2.31–3.20)	1.97 (1.67–2.32)	1.73 (1.46–2.04)	1.61 (1.35–1.92)
Patients with acute HMOD				
Tertile 1	REF	REF	REF	REF
Tertile 2	1.35 (1.06–1.73)	1.18 (0.93–1.51)	1.13 (0.88–1.45)	1.07 (0.83–1.38)
Tertile 3	2.79 (2.24–3.48)	2.25 (1.80–2.80)	2.11 (1.68–2.65)	1.96 (1.55–2.48)
Patients without acute HMOD				
Tertile 1	REF	REF	REF	REF
Tertile 2	1.32 (1.01–1.73)	1.16 (0.88–1.52)	1.12 (0.84–1.48)	1.09 (0.81–1.47)
Tertile 3	2.83 (2.22–3.61)	1.82 (1.42–2.33)	1.61 (1.25–2.08)	1.44 (1.10–1.88)

**Lymphocyte-to-monocyte ratio**	**Unadjusted HR (95% CI)**	**Model 1**^a^**(95% CI)**	**Model 2**^b^**(95% CI)**	**Model 3**^c^**(95% CI)**
All patients				
Tertile 1	REF	REF	REF	REF
Tertile 2	0.48 (0.41–0.55)	0.59 (0.50–0.68)	0.65 (0.55–0.76)	0.68 (0.58–0.80)
Tertile 3	0.39 (0.33–0.46)	0.61 (0.52–0.72)	0.68 (0.57–0.80)	0.71 (0.60–0.85)
Patients with acute HMOD				
Tertile 1	REF	REF	REF	REF
Tertile 2	0.52 (0.43–0.64)	0.60 (0.49–0.74)	0.65 (0.53–0.80)	0.70 (0.57–0.86)
Tertile 3	0.38 (0.31–0.48)	0.51 (0.41–0.64)	0.55 (0.44–0.69)	0.57 (0.45–0.72)
Patients without acute HMOD				
Tertile 1	REF	REF	REF	REF
Tertile 2	0.42 (0.34–0.53)	0.56 (0.44–0.70)	0.60 (0.48–0.76)	0.62 (0.48–0.79)
Tertile 3	0.40 (0.32–0.50)	0.72 (0.57–0.92)	0.77 (0.60–0.99)	0.85 (0.65–1.09)

**Platelet-to-lymphocyte ratio**	**Unadjusted HR (95% CI)**	**Model 1**^a^**(95% CI)**	**Model 2**^b^**(95% CI)**	**Model 3**^c^**(95% CI)**
All patients				
Tertile 1	REF	REF	REF	REF
Tertile 2	0.99 (0.83–1.17)	0.95 (0.79–1.13)	0.90 (0.75–1.08)	0.86 (0.71–1.03)
Tertile 3	1.96 (1.68–2.28)	1.52 (1.30–1.77)	1.36 (1.16–1.59)	1.22 (1.03–1.44)
Patients with acute HMOD				
Tertile 1	REF	REF	REF	REF
Tertile 2	1.05 (0.83–1.32)	1.00 (0.79–1.26)	0.96 (0.76–1.22)	0.96 (0.75–1.22)
Tertile 3	2.04 (1.66–2.50)	1.63 (1.33–2.01)	1.48 (1.19–1.83)	1.34 (1.07–1.67)
Patients without acute HMOD				
Tertile 1	REF	REF	REF	REF
Tertile 2	1.05 (0.81–1.37)	0.96 (0.74–1.25)	0.92 (0.70–1.21)	0.80 (0.60–1.07)
Tertile 3	2.19 (1.73–2.76)	1.56 (1.23–1.98)	1.43 (1.12–1.83)	1.21 (0.94–1.60)

**Systemic immune-inflammation index**	**Unadjusted HR (95% CI)**	**Model 1**^a^**(95% CI)**	**Model 2**^b^**(95% CI)**	**Model 3**^c^**(95% CI)**
All patients				
Tertile 1	REF	REF	REF	REF
Tertile 2	1.07 (0.90–1.28)	1.06 (0.89–1.26)	1.02 (0.85–1.22)	1.04 (0.86–1.25)
Tertile 3	2.16 (1.85–2.52)	1.83 (1.57–2.15)	1.66 (1.41–1.95)	1.57 (1.33–1.86)
Patients with acute HMOD				
Tertile 1	REF	REF	REF	REF
Tertile 2	1.16 (0.91–1.47)	1.20 (0.95–1.52)	1.19 (0.93–1.52)	1.17 (0.91–1.50)
Tertile 3	2.28 (1.85–2.82)	2.11 (1.71–2.61)	1.98 (1.59–2.46)	1.90 (1.51–2.37)
Patients without acute HMOD				
Tertile 1	REF	REF	REF	REF
Tertile 2	1.04 (0.80–1.36)	0.99 (0.76–1.29)	0.96 (0.73–1.26)	0.97 (0.73–1.29)
Tertile 3	2.19 (1.74–2.76)	1.65 (1.30–2.08)	1.53 (1.20–1.95)	1.37 (1.06–1.77)

**C-reactive protein**	**Unadjusted HR (95% CI)**	**Model 1**^a^**(95% CI)**	**Model 2**^b^**(95% CI)**	**Model 3**^c^**(95% CI)**
All patients				
Tertile 1	REF	REF	REF	REF
Tertile 2	1.40 (1.16–1.70)	1.29 (1.06–1.56)	1.28 (1.05–1.55)	1.28 (1.05–1.57)
Tertile 3	3.54 (3.00–4.18)	2.55 (2.16–3.02)	2.32 (1.95–2.76)	2.21 (1.84–2.65)
Patients with acute HMOD				
Tertile 1	REF	REF	REF	REF
Tertile 2	1.13 (0.87–1.45)	1.05 (0.81–1.35)	1.06 (0.82–1.37)	1.13 (0.86–1.48)
Tertile 3	2.94 (2.35–3.67)	2.23 (1.78–2.79)	2.14 (1.70–2.70)	2.14 (1.68–2.72)
Patients without acute HMOD				
Tertile 1	REF	REF	REF	REF
Tertile 2	1.63 (1.22–2.18)	1.52 (1.14–2.04)	1.52 (1.13–2.04)	1.44 (1.05–1.97)
Tertile 3	4.25 (3.30–5.47)	3.01 (2.33–3.88)	2.61 (2.01–3.37)	2.41 (1.83–3.17)

CI, Confidence Interval; CRP, C-Reactive Protein; ED, Emergency Department; HR, Hazard Ratio; LMR, Lymphocyte-to-Monocyte Ratio; NLR, Neutrophil-to-Lymphocyte Ratio; PLR, Platelet-to-Lymphocyte Ratio; REF, Reference; SII, Systemic Immune-Inflammation Index

a Model 1: Adjusted for age, sex, and systolic and diastolic blood pressure.

b Model 2: Adjusted for age, sex, systolic blood pressure, diastolic blood pressure, and comorbidities (hypertension, diabetes mellitus, dyslipidemia, ischemic stroke, hemorrhagic stroke, coronary artery disease, heart failure, and chronic kidney disease).

c Model 3: Adjusted for age, sex, systolic blood pressure, diastolic blood pressure, comorbidities (hypertension, diabetes mellitus, dyslipidemia, ischemic stroke, hemorrhagic stroke, coronary artery disease, heart failure, and chronic kidney disease), and components of hypertension-mediated organ damage (estimated glomerular filtration rate, hemoglobin level, cardiomegaly on chest radiography, left ventricular hypertrophy on electrocardiography, myocardial ischemia on electrocardiography, and atrial fibrillation on electrocardiography). The range of NLR in tertile 1 is < 2.04. The range of NLR in tertile 2 is 2.04–4.39. The range of NLR in tertile 3 is > 4.39. The range of LMR in tertile 1 is < 2.71. The range of LMR in tertile 2 is 2.71–4.40. The range of LMR in tertile 3 is > 4.40. The range of PLR in tertile 1 is < 95.08. The range of PLR in tertile 2 is 95.08–151.76. The range of PLR in tertile 3 is >151.76. The range of SII in tertile 1 is < 432.04. The range of SII in tertile 2 is 432.04–959.95. The range of SII in tertile 3 is > 959.95. The range of CRP in tertile 1 is < 0.12. The range of CRP in tertile 2 is 0.12–0.46. The range of CRP in tertile 3 is > 0.46.

Restricted cubic spline analyses revealed distinct dose-response relationships between systemic inflammatory indicators and all-cause mortality (Fig. 3). For NLR and SII, a linear increase in mortality risk was observed across their respective ranges (p for nonlinearity = 0.683 and 0.456, respectively), suggesting a dose-dependent positive association. In contrast, LMR showed a distinct inverse J-shaped relationship, with a sharp decrease in risk up to an LMR of approximately 4.0, followed by a gradual rebound at higher levels (p for nonlinearity < 0.001). PLR demonstrated a J-shaped association (p for nonlinearity < 0.001), where mortality risk initially decreased with increasing PLR, reaching a nadir near 150, and then progressively increased at higher values. CRP showed a nonlinear positive association (p for nonlinearity < 0.001) with a steep increase in risk at low concentrations of up to ∼4 mg/dL, after which the hazard plateaued.

**Fig. 3:**
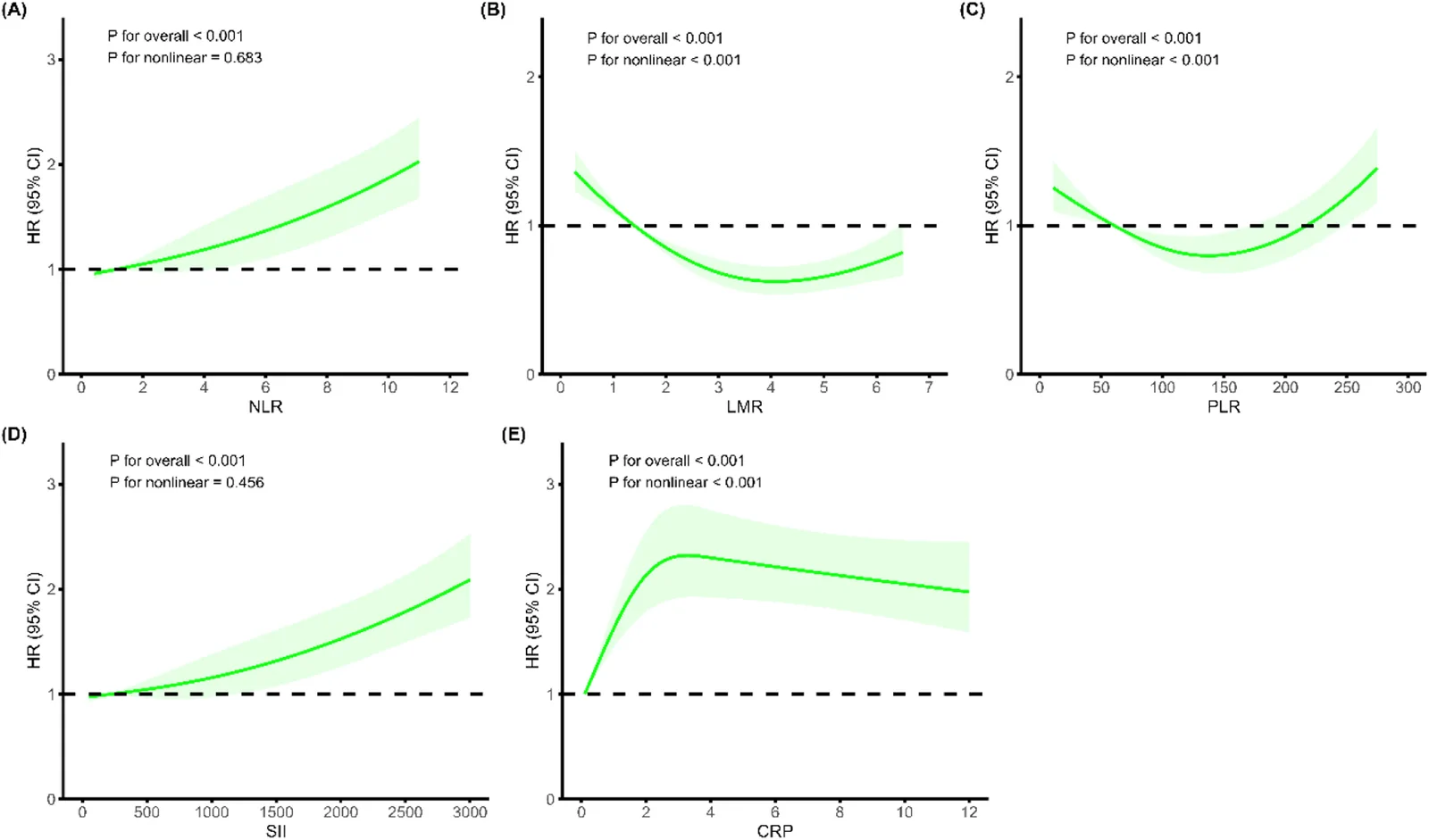
Restricted cubic spline curves for the association between systemic inflammatory indicators and all-cause mortality. (A) NLR, (B) LMR, (C) PLR, (D) SII, (E) CRP. CRP, C-Reactive Protein; LMR, Lymphocyte-to-Monocyte Ratio; NLR, Neutrophil-to-Lymphocyte Ratio; PLR, Platelet-to-Lymphocyte Ratio; SII, Systemic Immune-Inflammation Index. Adjusted for age, sex, systolic blood pressure, diastolic blood pressure, comorbidities (hypertension, diabetes mellitus, dyslipidemia, ischemic stroke, hemorrhagic stroke, coronary artery disease, heart failure, and chronic kidney disease), and components of subclinical hypertension-mediated organ damage (estimated glomerular filtration rate, hemoglobin level, cardiomegaly on chest radiography, and electrocardiographic findings such as left ventricular hypertrophy, myocardial ischemia, and atrial fibrillation).

### Predictive performance of systemic inflammatory indicators

We compared the time-dependent receiver operating characteristic curves for 3-year all-cause mortality to evaluate the prognostic utility of systemic inflammatory indicators (Fig. 4). CRP showed the highest AUC 0.677 (95% CI: 0.656–0.697), followed by NLR 0.646 (95% CI: 0.625–0.667), and LMR 0.639 (95% CI: 0.617–0.661). PLR 0.605 (95% CI: 0.582–0.628) and SII 0.614 (95% CI: 0.592–0.637) demonstrated lower predictive performance. CRP significantly outperformed the other indicators (all p < 0.01), whereas the predictive performance of NLR and LMR did not differ significantly (p = 0.450). The performances of PLR and SII were also comparable (p = 0.161).

**Fig. 4:**
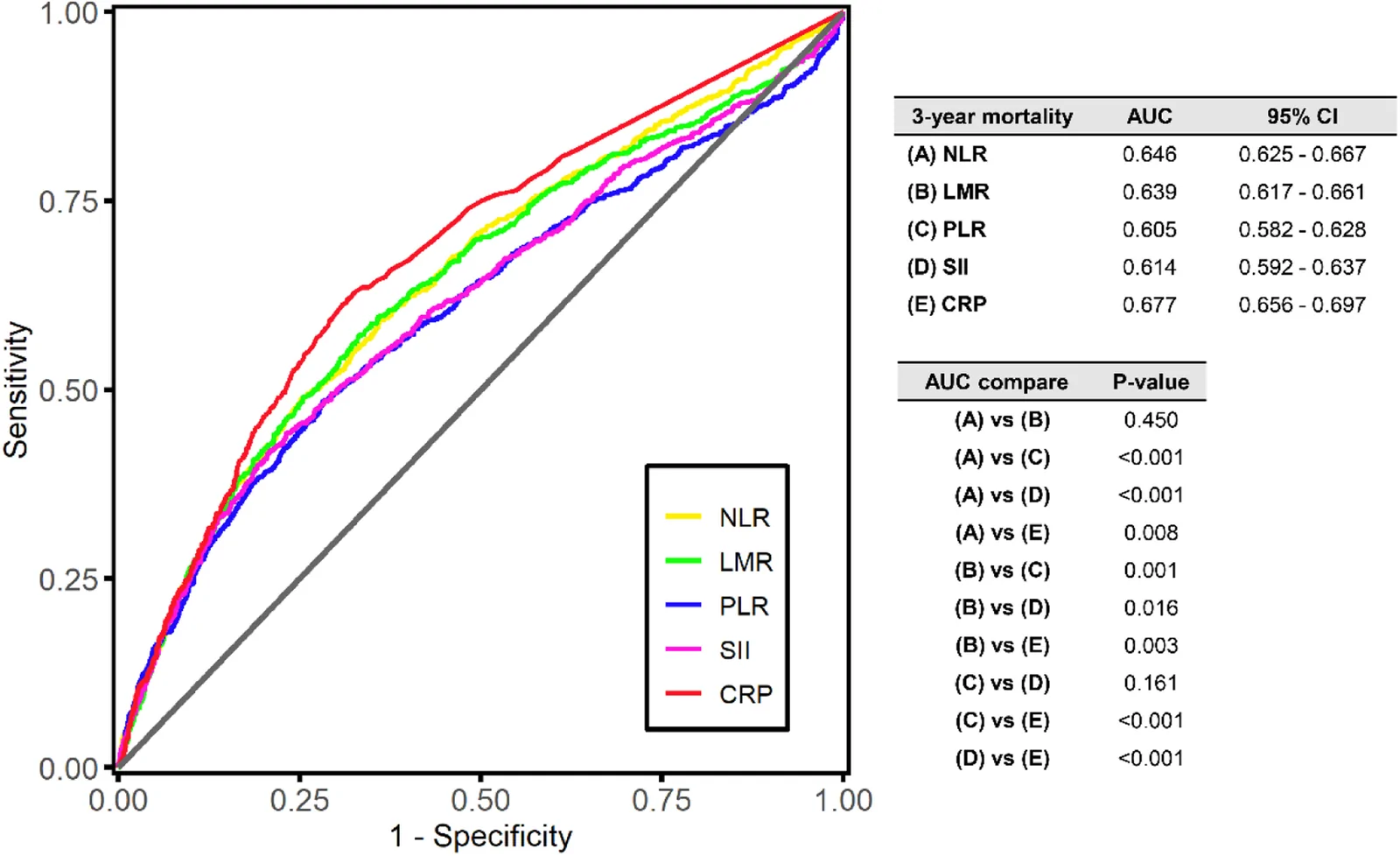
Time-dependent receiver operating characteristic curves for predicting 3-year all-cause mortality using systemic inflammatory indicators. AUC, Area Under the Curve; CI, Confidence Interval; CRP, C-Reactive Protein; LMR, Lymphocyte-to-Monocyte Ratio; NLR, Neutrophil-to-Lymphocyte Ratio; PLR, Platelet-to-Lymphocyte Ratio; SII, Systemic Immune-Inflammation Index.

## Discussion

This study evaluated the associations of systemic inflammatory indicators, including NLR, LMR, PLR, SII, and CRP, with long-term all-cause mortality in patients with severe hypertension and compared their discrimination for 3-year all-cause mortality. The key findings can be summarized as follows: 1) Elevated NLR, PLR, SII, and CRP levels were associated with an increased risk of all-cause mortality after multivariable adjustment, whereas elevated LMR was associated with a lower risk; 2) Dose-response analyses revealed linear positive associations for NLR and SII, an inverse J-shaped relationship for LMR, a J-shaped pattern for PLR, and a nonlinear positive association for CRP; and 3) CRP demonstrated the highest predictive performance for mortality, followed by NLR and LMR, although their overall discriminatory performance was modest.

Inflammation is increasingly recognized to play a critical role in the pathogenesis and progression of cardiovascular diseases,^[^^19^^]^ including hypertension.^[^^8^^]^ Neutrophils, lymphocytes, monocytes, and platelets orchestrate inflammatory responses and contribute to vascular remodeling, endothelial dysfunction, and HMOD.^[^^8^^]^ Previous studies have reported elevated inflammatory indicators in patients with severe hypertension, particularly in the presence of acute HMOD.^[^^9^^,^^20^^]^ Derhaschnig et al. demonstrated increased levels of inflammatory and endothelial activation markers in patients with hypertensive emergencies compared with those in urgent and normotensive controls.^[^^20^^]^ Similarly, Karabacak et al. showed that the levels of markers such as CRP and monocyte chemoattractant protein-1 were significantly higher in severe hypertension patients with HMOD than in those without.^[^^9^^]^ More recently, Duman Şahan et al. found that several systemic inflammatory indices, including NLR and systemic inflammation response index, were associated with in-hospital mortality among patients with hypertensive pulmonary edema.^[^^21^^]^ In a related acute cardiovascular setting, Scicchitano et al. reported that high-sensitivity C-reactive protein and NLR were associated with congestion and short-term mortality in patients with acute heart failure.^[^^22^^]^ These findings suggest that inflammatory markers measured during an acute presentation may reflect not only underlying systemic inflammation but also the severity of the acute cardiovascular condition. Nevertheless, data on the long-term prognostic significance of systemic inflammatory indicators in patients with severe hypertension remain limited.

In our study, all evaluated systemic inflammatory indicators, including NLR, LMR, PLR, SII, and CRP, were significantly associated with long-term all-cause mortality among patients with severe hypertension. This finding aligns with previous evidence that CRP is a well-established biomarker of cardiovascular risk, especially in atherosclerotic cardiovascular diseases.^[^^23^^]^ Its strong association with mortality in severe hypertension underscores its central role in systemic inflammation and vascular pathology.^[^^24^^]^ Notably, dose–response analyses revealed distinct patterns across biomarkers: NLR and SII demonstrated linear associations with increased mortality risk, whereas LMR exhibited an inverse J-shaped relationship. PLR showed a J-shaped pattern, and CRP displayed a nonlinear positive association with the risk, rising steeply at lower concentrations before reaching a plateau. A UK Biobank study conducted in a general population cohort reported a similar dose-response relationship for incident ischemic heart disease, stroke, and heart failure, with linear associations observed for NLR and SII, a nonlinear negative association for LMR, and a nonlinear positive association for CRP characterized by a steep risk increase at lower levels.^[^^10^^]^ These findings are broadly consistent with our results despite differences in population characteristics and clinical settings.

Several mechanisms have been proposed to explain the association between elevated levels of systemic inflammatory indicators and increased mortality during severe hypertension. First, higher systemic inflammatory states may exacerbate endothelial dysfunction, contributing to accelerated vascular injury, thrombosis, and subsequent cardiovascular events.^[^^8^^,^^25^^]^ Second, elevated inflammatory markers are often associated with a higher burden of comorbidities, including diabetes, CKD, coronary artery disease, and heart failure, all of which are independently associated with a poor prognosis.^[^^26^^]^ However, the associations in our study remained significant after adjustment for these comorbid conditions, although residual confounding cannot be excluded. Third, systemic inflammation might directly mediate or exacerbate HMOD, thereby amplifying adverse outcomes in acute settings.^[^^9^^,^^27^^]^ These findings highlight the need for further research to elucidate the underlying biological mechanisms and assess the potential utility of inflammatory markers in guiding risk stratification and management strategies for severe hypertension.

These findings may have clinical implications. CRP and NLR are readily available at ED presentation, and NLR can be calculated from a routine complete blood count without additional testing,^[^^28^^]^ making them practical and inexpensive for routine clinical use. Importantly, the associations were also observed in patients without acute HMOD, a group in whom long-term risk assessment can be challenging.^[^^29^^]^ These findings suggest that inflammatory markers may provide prognostic information in this population. However, their predictive performance was modest, and their role in risk stratification and follow-up planning requires further prospective evaluation.

This study had several limitations that warrant consideration. First, because this was a retrospective observational study based on NEDIS and electronic medical records, causal inference cannot be established. The retrospective design may also have introduced selection bias, particularly because inclusion in the final cohort required the availability of laboratory measurements needed to calculate the inflammatory indices. Clinical information collected during routine care may also have been incomplete or misclassified. Although trained research staff used predefined variable definitions and a standardized data abstraction form, formal interrater reliability testing was not performed. In addition, although the first available laboratory values obtained during the index ED visit were used, the exact time interval between the initial BP measurement and blood sampling was not available for all patients. Second, although derived from a large ED cohort, our data originated from a single regional emergency medical center in Korea, which potentially limits the generalizability of these findings. Third, we only analyzed baseline levels of inflammatory indicators and did not account for temporal changes, treatment interventions, or follow-up laboratory measurements that may affect long-term outcomes. Detailed information on pharmacological treatment before the index ED visit was not systematically available and therefore could not be included in the multivariable analyses. Fourth, despite multivariable adjustment, residual confounding cannot be completely excluded. Smoking status and BMI had substantial missing data and were therefore not included in the primary models. Although patients with infection recorded as an ED discharge diagnosis were excluded in a sensitivity analysis, infections already under treatment or not recorded at the ED visit, as well as chronic obstructive pulmonary disease and other concomitant inflammatory conditions, could not be fully identified. Finally, our dataset included only information on the death status and date, making it impossible to evaluate cause-specific mortality or adverse cardiovascular events. Nonetheless, the accuracy and completeness of the all-cause mortality data are considered high, given that the National Health Insurance Service database encompasses nearly the entire Korean population. Despite these limitations, this study has several strengths. The large ED cohort and long-term follow-up allowed for the parallel evaluation of multiple inflammatory markers. In addition, the dose–response analyses and comparison of predictive performance provided a more detailed assessment of their associations with long-term mortality.

## Conclusion

Systemic inflammatory indicators were associated with long-term mortality in patients with severe hypertension, with distinct dose-response patterns across markers. These readily available markers may provide prognostic information, although their role in clinical risk assessment requires further prospective evaluation.

## Statements

This study was reported in accordance with the Strengthening the Reporting of Observational Studies in Epidemiology (STROBE) guidelines.

## Declaration of generative AI and AI-assisted technologies in the writing process

During the preparation of this work, the authors used ChatGPT (OpenAI) for grammar and spelling checks. After using this tool, the authors reviewed and edited the content as needed and take full responsibility for the content of the publication.

## Authors’ contributions

Byungwoo Yoo: Writing-original draft; methodology; validation; investigation.

Byung Sik Kim: Conceptualization; writing-original draft; formal analysis; data curation.

Hyun-Jin Kim: Investigation; data curation; writing-review & editing.

Jeong-Hun Shin: Conceptualization; supervision; project administration; writing-review & editing.

## Funding

None.

## Data availability statement

Data will be shared on reasonable request to the corresponding author, if required.

## Declaration of competing interest

The authors declare no conflicts of interest.
