# Supplementary material for: Association between systemic inflammation and long-term mortality in emergency department patients with severe hypertension
Source: Clinics (Sao Paulo). 2026 Oct 3;81:101186. doi: 10.1016/j.clinsp.2026.101186 (PMC13653937; doi:10.1016/j.clinsp.2026.101186)
Supplement: Supplementary file 1 [file mmc1.docx]

**CLINICS-D-26-01896_Supplementary Material**

**Association between systemic inflammation and long-term mortality in emergency department patients with severe hypertension**

**Supplemental Table S1.** Missing rate for Covariates of Cox proportional hazards regression analyses

**Supplemental Table S2.** Comparison between the original data and imputed data

**Supplemental Table S3.** Baseline characteristics according to tertiles of lymphocyte-to-monocyte ratio

**Supplemental Table S4.** Baseline characteristics according to tertiles of platelet-to-lymphocyte ratio

**Supplemental Table S5.** Baseline characteristics according to tertiles of systemic immune-inflammation index

**Supplemental Table S6.** Baseline characteristics according to tertiles of C-reactive protein

**Supplemental Table S7.** Outcomes of the index visit to the emergency department and during the follow-up period according to tertiles of LMR, PLR, SII, and CRP levels

**Supplemental Table S8.** Sensitivity analysis comparing complete-case and multiple-imputation Cox regression results in all patients.

**Supplemental Table S9.** Sensitivity analysis further adjusting for smoking status and body mass index for 3-year all-cause mortality.

**Supplemental Table S10.** Sensitivity analysis after excluding patients with an infection diagnosis at the index emergency department visit.

**Supplemental Figure S1.** Kaplan–Meier curves for survival probability according to lymphocyte-to-monocyte ratio tertiles in (A) overall patients, (B) patients with acute HMOD, and (C) patients without acute HMOD. HMOD, hypertension-mediated organ damage.

**Supplemental Figure S2.** Kaplan–Meier curves for survival probability according to platelet-to-lymphocyte ratio tertiles in (A) overall patients, (B) patients with acute HMOD, and (C) patients without acute HMOD. HMOD, hypertension-mediated organ damage.

**Supplemental Figure S3.** Kaplan–Meier curves for survival probability according to systemic immune-inflammation index tertiles in (A) overall patients, (B) patients with acute HMOD, and (C) patients without acute HMOD. HMOD, hypertension-mediated organ damage.

**Supplemental Figure S4.** Kaplan–Meier curves for survival probability according to C-reactive protein tertiles in (A) overall patients, (B) patients with acute HMOD, and (C) patients without acute HMOD. HMOD, hypertension-mediated organ damage.

| **Supplemental Table S1.** Missing rate for Covariates of Cox proportional hazards regression analyses. | |  |
| --- | --- | --- |
| Covariates for Cox proportional hazards regression analyses | Missing value, n (%) | |
| Age | 0 | |
| Sex | 0 | |
| SBP | 0 | |
| DBP | 0 | |
| Hypertension | 128 (2.4) | |
| Diabetes mellitus | 174 (3.3) | |
| Dyslipidemia | 213 (4.0) | |
| Ischemic stroke | 212 (4.0) | |
| Hemorrhagic stroke | 221 (4.2) | |
| Coronary artery disease | 210 (3.9) | |
| Heart failure | 221 (4.2) | |
| Chronic kidney disease | 211 (4.0) | |
| eGFR | 20 (0.4) | |
| Hemoglobin | 5 (0.1) | |
| Cardiomegaly on chest radiography | 280 (5.3) | |
| Left ventricular hypertrophy on ECG | 624 (11.7) | |
| Myocardial ischemia on ECG | 624 (11.7) | |
| Atrial fibrillation on ECG | 624 (11.7) | |

DBP, diastolic blood pressure; ECG, electrocardiography; eGFR, estimated glomerular filtration rate; LVH, left ventricular hypertrophy; SBP, systolic blood pressure.

| **Supplemental Table S2.** Comparison between the original data and imputed data. | | | | |  |
| --- | --- | --- | --- | --- | --- |
| Covariates for Cox proportional hazards regression analyses | Original data  (n = 5,320) | Imputed data  (n = 5,320) | *p*-value^*^ | SMD | |
| Age, mean ± SD | 62.54 ± 16.60 | 62.54 ± 16.60 | 1 | 0 | |
| Women, *n* (%) | 2607 (49.0) | 2607 (49.0) | 1 | 0 | |
| SBP, mmHg, mean ± SD | 190.79 ± 22.14 | 190.79 ± 22.14 | 1 | 0 | |
| DBP, mmHg, mean ± SD | 107.64 ± 17.48 | 107.64 ± 17.48 | 1 | 0 | |
| Hypertension, *n* (%) | 2859 (55.1) | 2919 (54.9) | 0.87 | 0.004 | |
| Diabetes mellitus, *n* (%) | 1429 (27.8) | 1479 (27.8) | 0.937 | 0.002 | |
| Dyslipidemia, *n* (%) | 519 (10.2) | 549 (10.3) | 0.829 | 0.005 | |
| Ischemic stroke, *n* (%) | 430 (8.4) | 450 (8.5) | 0.918 | 0.003 | |
| Hemorrhagic stroke, *n* (%) | 150 (2.9) | 156 (2.9) | 0.933 | 0.003 | |
| Coronary artery disease, *n* (%) | 496 (9.7) | 516 (9.7) | 0.975 | 0.002 | |
| Heart failure, *n* (%) | 227 (4.5) | 238 (4.5) | 0.939 | 0.003 | |
| Chronic kidney disease, *n* (%) | 446 (8.7) | 455 (8.6) | 0.761 | 0.006 | |
| eGFR, mL/min/1.73 m^2^, mean ± SD | 79.44 ± 30.35 | 79.45 ± 30.34 | 0.979 | 0 | |
| Hemoglobin, g/dL, mean ± SD | 13.53 ± 2.17 | 13.53 ± 2.17 | 0.979 | 0.001 | |
| Cardiomegaly on chest radiography, *n* (%) | 711 (14.1) | 748 (14.1) | 0.935 | 0.003 | |
| Left ventricular hypertrophy on ECG, *n* (%) | 576 (12.3) | 640 (12) | 0.808 | 0.007 | |
| Myocardial ischemia on ECG, *n* (%) | 318 (6.8) | 356 (6.7) | 0.86 | 0.004 | |
| Atrial fibrillation on ECG, *n* (%) | 259 (5.5) | 283 (5.3) | 0.683 | 0.009 | |

DBP, diastolic blood pressure; ECG, electrocardiography; eGFR, estimated glomerular filtration rate; LVH, left ventricular hypertrophy; SBP, systolic blood pressure; SD, standard deviation; SMD, standardized mean differences.

^*^ Categorical variables were compared using the chi-square test or Fisher’s exact test, as appropriate. Continuous variables were compared using Student’s t-test.

| **Supplemental Table S3.** Baseline characteristics according to tertiles of lymphocyte-to-monocyte ratio. | | | | | |
| --- | --- | --- | --- | --- | --- |
|  | Lymphocyte-to-monocyte ratio | | | *p*-value |  |
|  | Tertile 1^*^  (*n* = 1,774) | Tertile 2^†^  (*n* = 1,773) | Tertile 3^‡^  (*n* = 1,773) |  |  |
| Age, mean | 65.71 ± 17.39 | 62.46 ± 16.21^a^ | 59.47 ± 15.58^ab^ | <0.001 |  |
| Women, *n* (%) | 756 (42.6) | 853 (48.1) | 998 (56.3) | <0.001 |  |
| Medical history, *n* (%) |  |  |  |  |  |
| Hypertension | 1051 (60.6) | 983 (56.8) | 825 (47.8) | <0.001 |  |
| Diabetes mellitus | 597 (34.8) | 477 (27.8) | 355 (20.7) | <0.001 |  |
| Dyslipidemia | 162 (9.5) | 183 (10.7) | 174 (10.2) | 0.503 |  |
| Ischemic stroke | 197 (11.6) | 134 (7.9) | 99 (5.8) | <0.001 |  |
| Hemorrhagic stroke | 52 (3.1) | 43 (2.5) | 55 (3.2) | 0.454 |  |
| Coronary artery disease | 172 (10.1) | 193 (11.3) | 131 (7.7) | 0.001 |  |
| Heart failure | 120 (7.1) | 67 (3.9) | 40 (2.4) | <0.001 |  |
| Chronic kidney disease | 245 (14.4) | 121 (7.1) | 80 (4.7) | <0.001 |  |
| End-stage renal disease | 124 (7.3) | 54 (3.2) | 38 (2.2) | <0.001 |  |
| Social history, *n* (%) |  |  |  |  |  |
| Cigarette smoking | 294 (23.3) | 323 (27.4) | 305 (26.5) | 0.102 |  |
| Alcohol consumption | 393 (30.7) | 444 (37.2) | 437 (37.4) | <0.001 |  |
| Triage vitals, mean |  |  |  |  |  |
| SBP, mmHg | 189.07 ± 22.21 | 191.73 ± 21.38^a^ | 191.55 ± 22.71^a^ | <0.001 |  |
| DBP, mmHg | 105.74 ± 18.07 | 107.50 ± 16.91^a^ | 109.69 ± 17.23^ab^ | <0.001 |  |
| Laboratory tests |  |  |  |  |  |
| eGFR, mL/min/1.73 m2 | 70.63 ± 34.04 | 81.44 ± 28.38^a^ | 86.24 ± 25.93^ab^ | <0.001 |  |
| Hb, g/dL | 13.06 ± 2.42 | 13.71 ± 2.03^a^ | 13.82 ± 1.94^a^ | <0.001 |  |
| BNP, pg/mL | 520.62 ± 939.73 | 307.54 ± 664.36^a^ | 277.15 ± 649.59^ab^ | <0.001^d^ |  |
| Troponin-I, ng/mL | 0.35 ± 3.93 | 0.10 ± 1.10^a^ | 0.08 ± 0.72^ab^ | <0.001^d^ |  |
| Neutrophil count, 10^9 cells/L | 8.50 ± 4.80 | 5.75 ± 2.89^a^ | 4.97 ± 2.83^ab^ | <0.001^d^ |  |
| Lymphocyte count, 10^9 cells/L | 1.21 ± 0.63 | 1.98 ± 0.80^a^ | 2.90 ± 3.18^ab^ | <0.001^d^ |  |
| Monocyte count, 10^9 cells/L | 0.75 ± 1.49 | 0.57 ± 0.22^a^ | 0.46 ± 0.22^ab^ | <0.001^d^ |  |
| Platelet count, 10^9 cells/L | 217.68 ± 84.69 | 224.69 ± 67.00^a^ | 229.85 ± 74.94^ab^ | <0.001^d^ |  |
| Urinary analysis |  |  |  |  |  |
| Proteinuria^c^, *n* (%) | 507 (41.7) | 321 (28.1) | 327 (28.6) | <0.001 |  |
| Chest radiography |  |  |  |  |  |
| Cardiomegaly, *n* (%) | 269 (16.0) | 246 (14.6) | 196 (11.7) | 0.002 |  |
| Electrocardiography |  |  |  |  |  |
| LVH, *n* (%) | 205 (13.0) | 205 (13.0) | 166 (10.7) | 0.08 |  |
| Myocardial ischemia, *n* (%) | 131 (8.3) | 85 (5.4) | 102 (6.6) | 0.004 |  |
| Atrial fibrillation, *n* (%) | 113 (7.2) | 78 (4.9) | 68 (4.4) | 0.001 |  |
| Acute HMOD, *n* (%) | 638 (36.0) | 615 (34.7) | 606 (34.2) | 0.517 |  |
| Systemic inflammation indicators |  |  |  |  |  |
| Neutrophil-to-lymphocyte ratio | 10.40 ± 13.47 | 3.41 ± 2.75^a^ | 2.39 ± 2.79^ab^ | <0.001^d^ |  |
| Lymphocyte-to-monocyte ratio | 1.75 ± 0.62 | 3.52 ± 0.48^a^ | 6.71 ± 4.43^ab^ | <0.001^d^ |  |
| Platelet-to-lymphocyte ratio | 237.19 ± 226.04 | 129.01 ± 67.42^a^ | 102.05 ± 73.77^ab^ | <0.001^d^ |  |
| Systemic immune inflammation index | 2355.23 ± 4002.81 | 769.72 ± 692.50^a^ | 552.32 ± 741.07^ab^ | <0.001^d^ |  |
| C-reactive protein, mg/dL | 3.51 ± 6.74 | 0.81 ± 2.71^a^ | 0.71 ± 2.63^ab^ | <0.001^d^ |  |

Data are presented as *n* (%) or mean ± SD, as appropriate. BNP, B-type natriuretic peptide; DBP, diastolic blood pressure; eGFR, estimated glomerular filtration rate; Hb, hemoglobin; HMOD, hypertension-mediated organ damage; LMR, lymphocyte-to-monocyte ratio; LVH, left ventricular hypertrophy; SD, standard deviation; SBP, systolic blood pressure.

Percentages were calculated using the number of patients with available data for each variable as the denominator.

^*^The range of LMR in tertile 1 is < 2.71.

^†^The range of LMR in tertile 2 is 2.71–4.40.

^‡^The range of LMR in tertile 3 is > 4.40.

^a^Post hoc p: Significant difference, p < 0.05, compared with the first tertile.

^b^Post hoc p: Significant difference, p < 0.05, compared with the second tertile.

^c^Proteinuria was defined as dipstick urinalysis result ≥ 1+.

^d^Assessed by nonparametric test

| **Supplemental Table S4.** Baseline characteristics according to tertiles of platelet-to-lymphocyte ratio. | | | | | |
| --- | --- | --- | --- | --- | --- |
|  | Platelet-to-lymphocyte ratio | | | *p*-value |  |
|  | Tertile 1^*^  (*n* = 1,774) | Tertile 2^†^  (*n* = 1,773) | Tertile 3^‡^  (*n* = 1,773) |  |  |
| Age, mean | 60.66 ± 15.73 | 61.42 ± 16.50^a^ | 65.55 ± 17.14^ab^ | <0.001 |  |
| Women, *n* (%) | 745 (42.0) | 902 (50.9) | 960 (54.1) | <0.001 |  |
| Medical history, *n* (%) |  |  |  |  |  |
| Hypertension | 883 (51.0) | 942 (54.4) | 1034 (59.8) | <0.001 |  |
| Diabetes mellitus | 400 (23.3) | 450 (26.3) | 579 (33.8) | <0.001 |  |
| Dyslipidemia | 183 (10.7) | 165 (9.7) | 171 (10.1) | 0.623 |  |
| Ischemic stroke | 101 (5.9) | 140 (8.2) | 189 (11.1) | <0.001 |  |
| Hemorrhagic stroke | 56 (3.3) | 42 (2.5) | 52 (3.1) | 0.355 |  |
| Coronary artery disease | 181 (10.6) | 161 (9.5) | 154 (9.0) | 0.291 |  |
| Heart failure | 63 (3.7) | 58 (3.4) | 106 (6.3) | <0.001 |  |
| Chronic kidney disease | 98 (5.7) | 125 (7.4) | 223 (13.1) | <0.001 |  |
| End-stage renal disease | 52 (3.0) | 59 (3.5) | 105 (6.2) | <0.001 |  |
| Social history, *n* (%) |  |  |  |  |  |
| Cigarette smoking | 376 (32.4) | 306 (25.7) | 240 (19.3) | <0.001 |  |
| Alcohol consumption | 506 (42.6) | 437 (36.4) | 331 (26.4) | <0.001 |  |
| Triage vitals, mean |  |  |  |  |  |
| SBP, mmHg | 192.97 ± 23.17 | 190.62 ± 21.41 | 188.76 ± 21.60^ab^ | <0.001 |  |
| DBP, mmHg | 109.07 ± 17.32 | 107.84 ± 16.96^a^ | 106.01 ± 18.02^ab^ | <0.001 |  |
| Laboratory tests |  |  |  |  |  |
| eGFR, mL/min/1.73 m2 | 82.85 ± 27.31 | 82.05 ± 29.06 | 73.40 ± 33.45^ab^ | <0.001 |  |
| Hb, g/dL | 14.03 ± 1.97 | 13.65 ± 2.01^a^ | 12.91 ± 2.35^ab^ | <0.001 |  |
| BNP, pg/mL | 316.10 ± 681.27 | 322.66 ± 679.31^a^ | 493.94 ± 942.90^ab^ | <0.001^d^ |  |
| Troponin-I, ng/mL | 0.11 ± 1.14 | 0.12 ± 0.89 | 0.31 ± 3.91^ab^ | <0.001^d^ |  |
| Neutrophil count, 10^9 cells/L | 5.15 ± 3.08 | 5.82 ± 3.03^a^ | 8.26 ± 4.72^ab^ | <0.001^d^ |  |
| Lymphocyte count, 10^9 cells/L | 3.14 ± 3.14 | 1.87 ± 0.56^a^ | 1.08 ± 0.47^ab^ | <0.001^d^ |  |
| Monocyte count, 10^9 cells/L | 0.68 ± 1.48 | 0.57 ± 0.25^a^ | 0.53 ± 0.28^ab^ | <0.001^d^ |  |
| Platelet count, 10^9 cells/L | 198.94 ± 64.48 | 222.81 ± 60.16^a^ | 250.48 ± 90.80^ab^ | <0.001^d^ |  |
| Urinary analysis |  |  |  |  |  |
| Proteinuria^c^, *n* (%) | 324 (28.5) | 324 (28.5) | 507 (41.2) | <0.001 |  |
| Chest radiography |  |  |  |  |  |
| Cardiomegaly, *n* (%) | 209 (12.5) | 251 (15.0) | 251 (14.9) | 0.065 |  |
| Electrocardiography |  |  |  |  |  |
| LVH, *n* (%) | 177 (11.3) | 187 (12.1) | 212 (13.4) | 0.2 |  |
| Myocardial ischemia, *n* (%) | 120 (7.7) | 92 (6.0) | 106 (6.7) | 0.167 |  |
| Atrial fibrillation, *n* (%) | 99 (6.3) | 71 (4.6) | 89 (5.6) | 0.108 |  |
| Acute HMOD, *n* (%) | 697 (39.3) | 574 (32.4) | 588 (33.2) | <0.001 |  |
| Systemic inflammation indicators |  |  |  |  |  |
| Neutrophil-to-lymphocyte ratio | 1.99 ± 2.19 | 3.41 ± 2.33^a^ | 10.80 ± 13.41^ab^ | <0.001^d^ |  |
| Lymphocyte-to-monocyte ratio | 5.28 ± 3.49 | 3.89 ± 2.65^a^ | 2.81 ± 3.26^ab^ | <0.001^d^ |  |
| Platelet-to-lymphocyte ratio | 69.16 ± 18.21 | 120.94 ± 15.98^a^ | 278.25 ± 216.87^ab^ | <0.001^d^ |  |
| Systemic immune inflammation index | 355.49 ± 244.12 | 705.47 ± 384.48^a^ | 2617.44 ± 3978.05^ab^ | <0.001^d^ |  |
| C-reactive protein, mg/dL | 0.75 ± 2.61 | 1.03 ± 2.77^a^ | 3.24 ± 6.82^ab^ | <0.001^d^ |  |

Data are presented as *n* (%) or mean ± SD, as appropriate. BNP, B-type natriuretic peptide; DBP, diastolic blood pressure; eGFR, estimated glomerular filtration rate; Hb, hemoglobin; HMOD, hypertension-mediated organ damage; LVH, left ventricular hypertrophy; PLR, platelet-to-lymphocyte ratio; SD, standard deviation; SBP, systolic blood pressure.

Percentages were calculated using the number of patients with available data for each variable as the denominator.

^*^The range of PLR in tertile 1 is < 95.08

^†^The range of PLR in tertile 2 is 95.08–151.76

^‡^The range of PLR in tertile 3 is >151.76

^a^Post hoc p: Significant difference, p < 0.05, compared with the first tertile

^b^Post hoc p: Significant difference, p < 0.05, compared with the second tertile

^c^Proteinuria was defined as dipstick urinalysis result ≥ 1+

^d^Assessed by nonparametric test

| **Supplemental Table S5.** Baseline characteristics according to tertiles of systemic immune-inflammation index. | | | | | |
| --- | --- | --- | --- | --- | --- |
|  | Systemic immune-inflammation index | | | *p*-value |  |
|  | Tertile 1^*^  (*n* = 1,774) | Tertile 2^†^  (*n* = 1,773) | Tertile 3^‡^  (*n* = 1,773) |  |  |
| Age, mean | 61.68 ± 15.46 | 61.47 ± 16.59 | 64.48 ± 17.53^ab^ | <0.001 |  |
| Women, *n* (%) | 847 (47.7) | 867 (48.9) | 893 (50.4) | 0.294 |  |
| Medical history, *n* (%) |  |  |  |  |  |
| Hypertension | 907 (52.3) | 955 (55.2) | 997 (57.7) | 0.005 |  |
| Diabetes mellitus | 378 (22.0) | 484 (28.2) | 567 (33.1) | <0.001 |  |
| Dyslipidemia | 185 (10.8) | 182 (10.7) | 152 (9.0) | 0.134 |  |
| Ischemic stroke | 110 (6.4) | 138 (8.1) | 182 (10.7) | <0.001 |  |
| Hemorrhagic stroke | 60 (3.5) | 39 (2.3) | 51 (3.0) | 0.107 |  |
| Coronary artery disease | 178 (10.4) | 175 (10.3) | 143 (8.4) | 0.085 |  |
| Heart failure | 45 (2.6) | 81 (4.8) | 101 (6.0) | <0.001 |  |
| Chronic kidney disease | 89 (5.2) | 147 (8.7) | 210 (12.4) | <0.001 |  |
| End-stage renal disease | 48 (2.8) | 70 (4.1) | 98 (5.8) | <0.001 |  |
| Social history, *n* (%) |  |  |  |  |  |
| Cigarette smoking | 297 (25.7) | 350 (29.3) | 275 (22.1) | 0.001 |  |
| Alcohol consumption | 479 (40.6) | 450 (37.4) | 345 (27.4) | <0.001 |  |
| Triage vitals, mean |  |  |  |  |  |
| SBP, mmHg | 191.63 ± 22.04 | 192.27 ± 22.37 | 188.46 ± 21.81^ab^ | <0.001 |  |
| DBP, mmHg | 108.12 ± 16.63 | 108.03 ± 17.83 | 106.78 ± 17.93^a^ | 0.038 |  |
| Laboratory tests |  |  |  |  |  |
| eGFR, mL/min/1.73 m2 | 83.82 ± 26.17 | 80.97 ± 30.17^a^ | 73.51 ± 33.35^ab^ | <0.001 |  |
| Hb, g/dL | 13.82 ± 1.97 | 13.64 ± 2.05^a^ | 13.13 ± 2.39^ab^ | <0.001 |  |
| BNP, pg/mL | 264.27 ± 615.43 | 353.46 ± 741.36^a^ | 515.87 ± 937.16^ab^ | <0.001^d^ |  |
| Troponin-I, ng/mL | 0.04 ± 0.20 | 0.11 ± 1.14^a^ | 0.40 ± 4.05^ab^ | <0.001^d^ |  |
| Neutrophil count, 10^9 cells/L | 3.76 ± 1.62 | 5.72 ± 2.02^a^ | 9.75 ± 4.57^ab^ | <0.001^d^ |  |
| Lymphocyte count, 10^9 cells/L | 2.88 ± 3.16 | 2.01 ± 0.89^a^ | 1.20 ± 0.62^ab^ | <0.001^d^ |  |
| Monocyte count, 10^9 cells/L | 0.60 ± 1.48 | 0.58 ± 0.27 | 0.60 ± 0.32 | 0.268^d^ |  |
| Platelet count, 10^9 cells/L | 197.28 ± 61.88 | 226.27 ± 65.10^a^ | 248.68 ± 89.20^ab^ | <0.001^d^ |  |
| Urinary analysis |  |  |  |  |  |
| Proteinuria^c^, *n* (%) | 264 (23.9) | 332 (29.0) | 559 (44.5) | <0.001 |  |
| Chest radiography |  |  |  |  |  |
| Cardiomegaly, *n* (%) | 240 (14.4) | 222 (13.2) | 249 (14.8) | 0.397 |  |
| Electrocardiography |  |  |  |  |  |
| LVH, *n* (%) | 178 (11.3) | 186 (11.9) | 212 (13.6) | 0.13 |  |
| Myocardial ischemia, *n* (%) | 99 (6.3) | 101 (6.5) | 118 (7.6) | 0.308 |  |
| Atrial fibrillation, *n* (%) | 87 (5.5) | 82 (5.3) | 90 (5.8) | 0.83 |  |
| Acute HMOD, *n* (%) | 652 (36.8) | 596 (33.6) | 611 (34.5) | 0.128 |  |
| Systemic inflammation indicators |  |  |  |  |  |
| Neutrophil-to-lymphocyte ratio | 1.53 ± 1.01 | 3.21 ± 1.99^a^ | 11.46 ± 13.17^ab^ | <0.001^d^ |  |
| Lymphocyte-to-monocyte ratio | 5.41 ± 3.87 | 3.90 ± 2.76^a^ | 2.67 ± 2.54^ab^ | <0.001^d^ |  |
| Platelet-to-lymphocyte ratio | 79.14 ± 30.95 | 124.49 ± 42.84^a^ | 264.72 ± 223.21^ab^ | <0.001^d^ |  |
| Systemic immune inflammation index | 276.94 ± 93.93 | 652.86 ± 146.68^a^ | 2748.64 ± 3926.68^ab^ | <0.001^d^ |  |
| C-reactive protein, mg/dL | 0.51 ± 1.72 | 1.03 ± 2.97^a^ | 3.48 ± 6.92^ab^ | <0.001^d^ |  |

Data are presented as *n* (%) or mean ± SD, as appropriate. BNP, B-type natriuretic peptide; DBP, diastolic blood pressure; eGFR, estimated glomerular filtration rate; Hb, hemoglobin; HMOD, hypertension-mediated organ damage; LVH, left ventricular hypertrophy; SII, systemic immune inflammation index; SD, standard deviation; SBP, systolic blood pressure.

Percentages were calculated using the number of patients with available data for each variable as the denominator.

^*^The range of SII in tertile 1 is < 432.04

^†^The range of SII in tertile 2 is 432.04–959.95

^‡^The range of SII in tertile 3 is > 959.95

^a^Post hoc p: Significant difference, p < 0.05, compared with the first tertile

^b^Post hoc p: Significant difference, p < 0.05, compared with the second tertile

^c^Proteinuria was defined as dipstick urinalysis result ≥ 1+

^d^Assessed by nonparametric test

| **Supplemental Table S6.** Baseline characteristics according to tertiles of C-reactive protein. | | | | | |
| --- | --- | --- | --- | --- | --- |
|  | C-reactive protein | | | *p*-value |  |
|  | Tertile 1^*^  (*n* = 1,774) | Tertile 2^†^  (*n* = 1,773) | Tertile 3^‡^  (*n* = 1,773) |  |  |
| Age, mean | 60.25 ± 15.82 | 61.47 ± 16.25 | 65.92 ± 17.19^ab^ | <0.001 |  |
| Women, *n* (%) | 924 (52.1) | 859 (48.4) | 824 (46.5) | 0.003 |  |
| Medical history, *n* (%) |  |  |  |  |  |
| Hypertension | 928 (53.4) | 916 (52.6) | 1015 (59.3) | <0.001 |  |
| Diabetes mellitus | 425 (24.5) | 427 (24.8) | 577 (34.1) | <0.001 |  |
| Dyslipidemia | 200 (11.6) | 166 (9.7) | 153 (9.2) | 0.043 |  |
| Ischemic stroke | 105 (6.1) | 127 (7.4) | 198 (11.9) | <0.001 |  |
| Hemorrhagic stroke | 49 (2.8) | 43 (2.5) | 58 (3.5) | 0.233 |  |
| Coronary artery disease | 169 (9.8) | 158 (9.2) | 169 (10.1) | 0.654 |  |
| Heart failure | 45 (2.6) | 55 (3.2) | 127 (7.6) | <0.001 |  |
| Chronic kidney disease | 108 (6.3) | 99 (5.8) | 239 (14.3) | <0.001 |  |
| End-stage renal disease | 46 (2.7) | 44 (2.6) | 126 (7.6) | <0.001 |  |
| Social history, *n* (%) |  |  |  |  |  |
| Cigarette smoking | 314 (27.1) | 329 (28.9) | 279 (21.5) | <0.001 |  |
| Alcohol consumption | 472 (40.1) | 442 (38.0) | 360 (27.6) | <0.001 |  |
| Triage vitals, mean |  |  |  |  |  |
| SBP, mmHg | 191.06 ± 20.52 | 192.07 ± 22.87 | 189.23 ± 22.85^ab^ | 0.001 |  |
| DBP, mmHg | 107.34 ± 16.53 | 108.59 ± 17.07 | 107.00 ± 18.74^b^ | 0.018 |  |
| Laboratory tests |  |  |  |  |  |
| eGFR, mL/min/1.73 m2 | 83.83 ± 27.85 | 83.58 ± 27.05 | 70.90 ± 33.87^ab^ | <0.001 |  |
| Hb, g/dL | 13.73 ± 1.95 | 13.91 ± 1.98^a^ | 12.95 ± 2.43^ab^ | <0.001 |  |
| BNP, pg/mL | 226.91 ± 564.52 | 262.16 ± 596.27^a^ | 587.25 ± 988.38^ab^ | <0.001^d^ |  |
| Troponin-I, ng/mL | 0.06 ± 0.91 | 0.10 ± 0.69^a^ | 0.38 ± 4.02^ab^ | <0.001^d^ |  |
| Neutrophil count, 10^9 cells/L | 5.45 ± 3.14 | 5.70 ± 3.03^a^ | 8.08 ± 4.80^ab^ | <0.001^d^ |  |
| Lymphocyte count, 10^9 cells/L | 2.22 ± 1.15 | 2.20 ± 1.21 | 1.67 ± 3.10^ab^ | <0.001^d^ |  |
| Monocyte count, 10^9 cells/L | 0.53 ± 0.21 | 0.57 ± 0.25^a^ | 0.68 ± 1.50^ab^ | <0.001^d^ |  |
| Platelet count, 10^9 cells/L | 225.43 ± 66.24 | 222.83 ± 69.44 | 223.96 ± 90.22^a^ | 0.044^d^ |  |
| Urinary analysis |  |  |  |  |  |
| Proteinuria^c^, *n* (%) | 273 (24.5) | 303 (27.2) | 579 (45.4) | <0.001 |  |
| Chest radiography |  |  |  |  |  |
| Cardiomegaly, *n* (%) | 201 (12.0) | 242 (14.4) | 268 (15.8) | 0.007 |  |
| Electrocardiography |  |  |  |  |  |
| LVH, *n* (%) | 188 (12.2) | 182 (11.7) | 206 (13.0) | 0.533 |  |
| Myocardial ischemia, *n* (%) | 87 (5.6) | 99 (6.3) | 132 (8.3) | 0.008 |  |
| Atrial fibrillation, *n* (%) | 45 (2.9) | 103 (6.6) | 111 (7.0) | <0.001 |  |
| Acute HMOD, *n* (%) | 564 (31.8) | 658 (37.1) | 637 (35.9) | 0.002 |  |
| Systemic inflammation indicators |  |  |  |  |  |
| Neutrophil-to-lymphocyte ratio | 3.42 ± 4.25 | 3.72 ± 4.23^a^ | 9.06 ± 13.37^ab^ | <0.001^d^ |  |
| Lymphocyte-to-monocyte ratio | 4.54 ± 2.68 | 4.22 ± 2.31^a^ | 3.22 ± 4.40^ab^ | <0.001^d^ |  |
| Platelet-to-lymphocyte ratio | 127.17 ± 88.62 | 129.02 ± 84.04 | 212.11 ± 227.42^ab^ | <0.001^d^ |  |
| Systemic immune inflammation index | 778.36 ± 1097.45 | 825.14 ± 1013.99^a^ | 2074.66 ± 3960.31^ab^ | <0.001^d^ |  |
| C-reactive protein, mg/dL | 0.10 ± 0.01 | 0.24 ± 0.09^a^ | 4.68 ± 7.15^ab^ | <0.001^d^ |  |

Data are presented as *n* (%) or mean ± SD, as appropriate. BNP, B-type natriuretic peptide; CRP, C-reactive protein; DBP, diastolic blood pressure; eGFR, estimated glomerular filtration rate; Hb, hemoglobin; HMOD, hypertension-mediated organ damage; LVH, left ventricular hypertrophy; SD, standard deviation; SBP, systolic blood pressure.

Percentages were calculated using the number of patients with available data for each variable as the denominator.

^*^The range of CRP in tertile 1 is <0.12

^†^The range of CRP in tertile 2 is 0.12–0.46

^‡^The range of CRP in tertile 3 is >0.46

^a^Post hoc p: Significant difference, p <0.05, compared with the first tertile

^b^Post hoc p: Significant difference, p <0.05, compared with the second tertile

^c^Proteinuria was defined as dipstick urinalysis result ≥1+

^d^Assessed by nonparametric test

| **Supplemental Table S7.** Outcomes of the index visit to the emergency department and during the follow-up period according to tertiles of LMR, PLR, SII, and CRP levels. | | | | | |
| --- | --- | --- | --- | --- | --- |
| **Lymphocyte-to-monocyte ratio** | Tertile 1  (*n* = 1,774) | Tertile 2  (*n* = 1,773) | Tertile 3  (*n* = 1,773) | *p*-value |  |
| Outcomes of the index visit to the ED, *n* (%) |  |  |  |  |  |
| Admission | 1,083 (61.0) | 868 (49.0) | 867 (48.9) | <0.001 |  |
| Discharge | 513 (28.9) | 722 (40.7) | 736 (41.5) | <0.001 |  |
| Discharge against medical advice | 176 (9.9) | 182 (10.3) | 167 (9.4) | 0.697 |  |
| Death in the emergency department | 2 (0.1) | 1 (0.1) | 3 (0.2) | 0.606 |  |
| Revisit to ED, *n* (%) |  |  |  |  |  |
| 1-month revisit | 138 (7.8) | 129 (7.3) | 113 (6.4) | 0.258 |  |
| 3-months revisit | 258 (14.5) | 217 (12.2) | 220 (12.4) | 0.076 |  |
| 1-year revisit | 438 (24.7) | 413 (23.3) | 359 (20.2) | 0.005 |  |
| Mortality, *n* (%) |  |  |  |  |  |
| 1-month mortality | 111 (6.3) | 53 (3.0) | 52 (2.9) | <0.001 |  |
| 3-months mortality | 177 (10.0) | 73 (4.1) | 81 (4.6) | <0.001 |  |
| 1-year mortality | 332 (18.7) | 150 (8.5) | 138 (7.8) | <0.001 |  |
| 3-year mortality | 494 (27.8) | 257 (14.5) | 214 (12.1) | <0.001 |  |
| **Platelet-to-lymphocyte ratio** | Tertile 1  (*n* = 1,774) | Tertile 2  (*n* = 1,773) | Tertile 3  (*n* = 1,773) | *p*-value |  |
| Outcomes of the index visit to the ED, *n* (%) |  |  |  |  |  |
| Admission | 922 (52.0) | 858 (48.4) | 1038 (58.5) | <0.001 |  |
| Discharge | 670 (37.8) | 729 (41.1) | 572 (32.3) | <0.001 |  |
| Discharge against medical advice | 180 (10.1) | 184 (10.4) | 161 (9.1) | 0.385 |  |
| Death in the emergency department | 2 (0.1) | 2 (0.1) | 2 (0.1) | 1 |  |
| Revisit to ED, *n* (%) |  |  |  |  |  |
| 1-month revisit | 129 (7.3) | 122 (6.9) | 129 (7.3) | 0.872 |  |
| 3-months revisit | 245 (13.8) | 208 (11.7) | 242 (13.6) | 0.124 |  |
| 1-year revisit | 421 (23.7) | 381 (21.5) | 408 (23.0) | 0.266 |  |
| Mortality, *n* (%) |  |  |  |  |  |
| 1-month mortality | 71 (4.0) | 45 (2.5) | 100 (5.6) | <0.001 |  |
| 3-months mortality | 100 (5.6) | 74 (4.2) | 157 (8.9) | <0.001 |  |
| 1-year mortality | 169 (9.5) | 151 (8.5) | 300 (16.9) | <0.001 |  |
| 3-year mortality | 254 (14.3) | 252 (14.2) | 459 (25.9) | <0.001 |  |
| **Systemic immune inflammation index** | Tertile 1  (*n* = 1,774) | Tertile 2  (*n* = 1,773) | Tertile 3  (*n* = 1,773) | *p*-value |  |
| Outcomes of the index visit to the ED, *n* (%) |  |  |  |  |  |
| Admission | 852 (48.0) | 861 (48.6) | 1105 (62.3) | <0.001 |  |
| Discharge | 730 (41.1) | 732 (41.3) | 509 (28.7) | <0.001 |  |
| Discharge against medical advice | 191 (10.8) | 177 (10.0) | 157 (8.9) | 0.159 |  |
| Death in the emergency department | 1 (0.1) | 3 (0.2) | 2 (0.1) | 0.606 |  |
| Revisit to ED, *n* (%) |  |  |  |  |  |
| 1-month revisit | 119 (6.7) | 143 (8.1) | 118 (6.7) | 0.181 |  |
| 3-months revisit | 236 (13.3) | 234 (13.2) | 225 (12.7) | 0.846 |  |
| 1-year revisit | 421 (23.7) | 407 (23.0) | 382 (21.5) | 0.289 |  |
| Mortality, *n* (%) |  |  |  |  |  |
| 1-month mortality | 49 (2.8) | 55 (3.1) | 112 (6.3) | <0.001 |  |
| 3-months mortality | 77 (4.3) | 80 (4.5) | 174 (9.8) | <0.001 |  |
| 1-year mortality | 146 (8.2) | 159 (9.0) | 315 (17.8) | <0.001 |  |
| 3-year mortality | 239 (13.5) | 255 (14.4) | 471 (26.6) | <0.001 |  |
| **C-reactive protein** | Tertile 1^*^  (*n* = 1,774) | Tertile 2^†^  (*n* = 1,773) | Tertile 3^‡^  (*n* = 1,773) | *p*-value |  |
| Outcomes of the index visit to the ED, n (%) |  |  |  |  |  |
| Admission | 757 (42.7) | 897 (50.6) | 1164 (65.7) | <0.001 |  |
| Discharge | 820 (46.2) | 684 (38.6) | 467 (26.3) | <0.001 |  |
| Discharge against medical advice | 196 (11.0) | 191 (10.8) | 138 (7.8) | 0.001 |  |
| Death in the emergency department | 1 (0.1) | 1 (0.1) | 4 (0.2) | 0.223 |  |
| Revisit to ED, n (%) |  |  |  |  |  |
| 1-month revisit | 119 (6.7) | 126 (7.1) | 135 (7.6) | 0.576 |  |
| 3-months revisit | 207 (11.7) | 230 (13.0) | 258 (14.6) | 0.039 |  |
| 1-year revisit | 374 (21.1) | 402 (22.7) | 434 (24.5) | 0.054 |  |
| Mortality, *n* (%) |  |  |  |  |  |
| 1-month mortality | 23 (1.3) | 49 (2.8) | 144 (8.1) | <0.001 |  |
| 3-months mortality | 40 (2.3) | 70 (3.9) | 221 (12.5) | <0.001 |  |
| 1-year mortality | 95 (5.4) | 142 (8.0) | 383 (21.6) | <0.001 |  |
| 3-year mortality | 187 (10.5) | 239 (13.5) | 539 (30.4) | <0.001 |  |

Data are presented as *n* (%). CRP, C-reactive protein; ED, emergency department; LMR, lymphocyte-to-monocyte ratio; PLR, platelet-to-lymphocyte ratio; SII, systemic immune inflammation index

The range of LMR in tertile 1 is <2.71

The range of LMR in tertile 2 is 2.71–4.40

The range of LMR in tertile 3 is >4.40

The range of PLR in tertile 1 is <95.08

The range of PLR in tertile 2 is 95.08–151.76

The range of PLR in tertile 3 is >151.76

The range of SII in tertile 1 is <432.04

The range of SII in tertile 2 is 432.04–959.95

The range of SII in tertile 3 is >959.95

The range of CRP in tertile 1 is <0.12

The range of CRP in tertile 2 is 0.12–0.46

The range of CRP in tertile 3 is >0.46

| **Supplemental Table S8.** Sensitivity analysis comparing complete-case and multiple-imputation Cox regression results in all patients. | | |
| --- | --- | --- |
|  | **Complete-case HR (95% CI)** | **Multiple-imputation HR (95% CI)** |
| **Neutrophil-to-lymphocyte ratio** |  |  |
| Tertile 1 | REF | REF |
| Tertile 2 | 1.06 (0.88–1.29) | 1.06 (0.88-1.27) |
| Tertile 3 | 1.61 (1.35–1.92) | 1.59 (1.34-1.88) |
| **Lymphocyte-to-monocyte ratio** |  |  |
| Tertile 1 | REF | REF |
| Tertile 2 | 0.68 (0.58–0.80) | 0.69 (0.59-0.81) |
| Tertile 3 | 0.71 (0.60–0.85) | 0.74 (0.63-0.88) |
| **Platelet-to-lymphocyte ratio** |  |  |
| Tertile 1 | REF | REF |
| Tertile 2 | 0.86 (0.71–1.03) | 0.88 (0.74-1.04) |
| Tertile 3 | 1.22 (1.03–1.44) | 1.23 (1.05-1.45) |
| **Systemic immune inflammation index** |  |  |
| Tertile 1 | REF | REF |
| Tertile 2 | 1.04 (0.86–1.25) | 0.99 (0.83-1.18) |
| Tertile 3 | 1.57 (1.33–1.86) | 1.54 (1.31-1.80) |
| **C-reactive protein** |  |  |
| Tertile 1 | REF | REF |
| Tertile 2 | 1.28 (1.05–1.57) | 1.26 (1.04-1.52) |
| Tertile 3 | 2.21 (1.84–2.65) | 2.12 (1.79-2.51) |

Values are hazard ratios with 95% confidence intervals for 3-year all-cause mortality. Complete-case results correspond to the Model 3 results presented in Table 3. Multiple-imputation analyses were performed among the 5,320 patients included in the final study cohort, with missing covariate data imputed using multiple imputation by chained equations. The same covariates as those in the primary Model 3 analysis were used for each inflammatory marker.

| **Supplemental Table S9.** Sensitivity analysis further adjusting for smoking status and body mass index for 3-year all-cause mortality. | | | | |
| --- | --- | --- | --- | --- |
|  | **Model 3**  **HR (95% CI)** **(n = 4,402)** | **Model 3 + smoking HR (95% CI)** **(n = 3,223)** | **Model 3 + BMI**  **HR (95% CI)** **(n = 2,736)** | **Model 3 + smoking + BMI**  **HR (95% CI)** **(n = 2,718)** |
| **Neutrophil-to-lymphocyte ratio** | |  |  |  |
| Tertile 1 | REF | REF | REF | REF |
| Tertile 2 | 1.06 (0.88–1.29) | 1.07 (0.86–1.34) | 1.04 (0.82–1.33) | 1.05 (0.83–1.34) |
| Tertile 3 | 1.61 (1.35–1.92) | 1.70 (1.38–2.09) | 1.63 (1.31–2.03) | 1.66 (1.33–2.07) |
| **Lymphocyte-to-monocyte ratio** | |  |  |  |
| Tertile 1 | REF | REF | REF | REF |
| Tertile 2 | 0.68 (0.58–0.80) | 0.63 (0.53–0.76) | 0.67 (0.55–0.81) | 0.66 (0.55–0.80) |
| Tertile 3 | 0.71 (0.60–0.85) | 0.69 (0.56–0.84) | 0.69 (0.55–0.85) | 0.68 (0.55–0.85) |
| **Platelet-to-lymphocyte ratio** | |  |  |  |
| Tertile 1 | REF | REF | REF | REF |
| Tertile 2 | 0.86 (0.71–1.03) | 0.91 (0.73–1.12) | 0.96 (0.77–1.20) | 0.97 (0.77–1.21) |
| Tertile 3 | 1.22 (1.03–1.44) | 1.23 (1.02–1.49) | 1.20 (0.98–1.47) | 1.22 (0.99–1.49) |
| **Systemic immune-inflammation index** | |  |  |  |
| Tertile 1 | REF | REF | REF | REF |
| Tertile 2 | 1.04 (0.86–1.25) | 0.96 (0.78–1.19) | 0.98 (0.78–1.23) | 0.98 (0.78–1.23) |
| Tertile 3 | 1.57 (1.33–1.86) | 1.57 (1.29–1.90) | 1.50 (1.23–1.84) | 1.54 (1.25–1.89) |
| **C-reactive protein** | |  |  |  |
| Tertile 1 | REF | REF | REF | REF |
| Tertile 2 | 1.28 (1.05–1.57) | 1.26 (0.99–1.59) | 1.14 (0.89–1.47) | 1.14 (0.89–1.47) |
| Tertile 3 | 2.21 (1.84–2.65) | 2.22 (1.80–2.74) | 2.08 (1.67–2.58) | 2.10 (1.69–2.62) |

Values are hazard ratios with 95% confidence intervals for 3-year all-cause mortality. Model 3 was adjusted for age, sex, systolic blood pressure, diastolic blood pressure, hypertension, diabetes mellitus, dyslipidemia, ischemic stroke, hemorrhagic stroke, coronary artery disease, heart failure, chronic kidney disease, estimated glomerular filtration rate, hemoglobin, cardiomegaly on chest radiography, left ventricular hypertrophy, myocardial ischemia, and atrial fibrillation on electrocardiography. Smoking status and body mass index (BMI) were additionally included in the corresponding sensitivity models. The number of participants differed across models because smoking status and BMI were missing in a substantial proportion of patients.

| **Supplemental Table S10.** Sensitivity analysis after excluding patients with an infection diagnosis at the index emergency department visit. | | |
| --- | --- | --- |
|  | **Model 3**  **HR (95% CI) (n = 4,402)** | **Model 3**  **(after excluding infection)**  **HR (95% CI) (n = 4,002)** |
| **Neutrophil-to-lymphocyte ratio** |  |  |
| Tertile 1 | REF | REF |
| Tertile 2 | 1.06 (0.88–1.29) | 1.06 (0.87–1.29) |
| Tertile 3 | 1.61 (1.35–1.92) | 1.73 (1.43–2.08) |
| **Lymphocyte-to-monocyte ratio** |  |  |
| Tertile 1 | REF | REF |
| Tertile 2 | 0.68 (0.58–0.80) | 0.66 (0.55–0.78) |
| Tertile 3 | 0.71 (0.60–0.85) | 0.64 (0.53–0.77) |
| **Platelet-to-lymphocyte ratio** |  |  |
| Tertile 1 | REF | REF |
| Tertile 2 | 0.86 (0.71–1.03) | 0.85 (0.70–1.03) |
| Tertile 3 | 1.22 (1.03–1.44) | 1.26 (1.06–1.50) |
| **Systemic immune inflammation index** |  |  |
| Tertile 1 | REF | REF |
| Tertile 2 | 1.04 (0.86–1.25) | 1.03 (0.85–1.25) |
| Tertile 3 | 1.57 (1.33–1.86) | 1.68 (1.41–2.00) |
| **C-reactive protein** |  |  |
| Tertile 1 | REF | REF |
| Tertile 2 | 1.28 (1.05–1.57) | 1.33 (1.08–1.64) |
| Tertile 3 | 2.21 (1.84–2.65) | 2.38 (1.96–2.87) |

Values are hazard ratios with 95% confidence intervals for 3-year all-cause mortality. Of the 496 patients with an infection diagnosis in the overall cohort, 400 were included in the primary complete-case Model 3 and were therefore excluded from this sensitivity analysis. The same covariates as those in the primary Model 3 analysis were used.

**Supplemental Figure S1.** Kaplan–Meier curves for survival probability according to lymphocyte-to-monocyte ratio tertiles in (A) overall patients, (B) patients with acute HMOD, and (C) patients without acute HMOD. HMOD, hypertension-mediated organ damage.

**Supplemental Figure S2.** Kaplan–Meier curves for survival probability according to platelet-to-lymphocyte ratio tertiles in (A) overall patients, (B) patients with acute HMOD, and (C) patients without acute HMOD. HMOD, hypertension-mediated organ damage.

**Supplemental Figure S3.** Kaplan–Meier curves for survival probability according to systemic immune-inflammation index tertiles in (A) overall patients, (B) patients with acute HMOD, and (C) patients without acute HMOD. HMOD, hypertension-mediated organ damage.

**Supplemental Figure S4.** Kaplan–Meier curves for survival probability according to C-reactive protein tertiles in (A) overall patients, (B) patients with acute HMOD, and (C) patients without acute HMOD. HMOD, hypertension-mediated organ damage
